# A novel spatiotemporal graph convolutional network framework for functional connectivity biomarkers identification of Alzheimer’s disease

**DOI:** 10.1186/s13195-024-01425-8

**Published:** 2024-03-14

**Authors:** Ying Zhang, Le Xue, Shuoyan Zhang, Jiacheng Yang, Qi Zhang, Min Wang, Luyao Wang, Mingkai Zhang, Jiehui Jiang, Yunxia Li, for the Alzheimer’s Disease Neuroimaging Initiative, Michael W. Weiner, Paul Aisen, Ronald Petersen, Clifford R. Jack, William Jagust, John Q. Trojanowski, Arthur W. Toga, Laurel Beckett, Robert C. Green, Andrew J. Saykin, John Morris, Leslie M. Shaw, Zaven Khachaturian, Greg Sorensen, Lew Kuller, Marcus Raichle, Steven Paul, Peter Davies, Howard Fillit, Franz Hefti, David Holtzman, Marek M. Mesulam, William Potter, Peter Snyder, Adam Schwartz, Tom Montine, Ronald G. Thomas, Michael Donohue, Sarah Walter, Devon Gessert, Tamie Sather, Gus Jiminez, Danielle Harvey, Matthew Bernstein, Paul Thompson, Norbert Schuff, Bret Borowski, Jeff Gunter, Matt Senjem, Prashanthi Vemuri, David Jones, Kejal Kantarci, Chad Ward, Robert A. Koeppe, Norm Foster, Eric M. Reiman, Kewei Chen, Chet Mathis, Susan Landau, Nigel J. Cairns, Erin Householder, Lisa Taylor-Reinwald, Virginia Lee, Magdalena Korecka, Michal Figurski, Karen Crawford, Scott Neu, Tatiana M. Foroud, Steven G. Potkin, Li Shen, Kelley Faber, Sungeun Kim, Kwangsik Nho, Leon Thal, Neil Buckholtz, Marylyn Albert, Richard Frank, John Hsiao, Jeffrey Kaye, Joseph Quinn, Betty Lind, Raina Carter, Sara Dolen, Lon S. Schneider, Sonia Pawluczyk, Mauricio Beccera, Liberty Teodoro, Bryan M. Spann, James Brewer, Helen Vanderswag, Adam Fleisher, Judith L. Heidebrink, Joanne L. Lord, Sara S. Mason, Colleen S. Albers, David Knopman, Kris Johnson, Rachelle S. Doody, Javier Villanueva-Meyer, Munir Chowdhury, Susan Rountree, Mimi Dang, Yaakov Stern, Lawrence S. Honig, Karen L. Bell, Beau Ances, Maria Carroll, Sue Leon, Mark A. Mintun, Stacy Schneider, Angela Oliver, Daniel Marson, Randall Griffith, David Clark, David Geldmacher, John Brockington, Erik Roberson, Hillel Grossman, Effie Mitsis, Leyla de Toledo-Morrell, Raj C. Shah, Ranjan Duara, Daniel Varon, Maria T. Greig, Peggy Roberts, Chiadi Onyike, Daniel D’Agostino, Stephanie Kielb, James E. Galvin, Brittany Cerbone, Christina A. Michel, Henry Rusinek, Mony J. de Leon, Lidia Glodzik, Susan De Santi, PMurali Doraiswamy, Jeffrey R. Petrella, Terence Z. Wong, Steven E. Arnold, Jason H. Karlawish, David Wolk, Charles D. Smith, Greg Jicha, Peter Hardy, Partha Sinha, Elizabeth Oates, Gary Conrad, Oscar L. Lopez, MaryAnn Oakley, Donna M. Simpson, Anton P. Porsteinsson, Bonnie S. Goldstein, Kim Martin, Kelly M. Makino, MSaleem Ismail, Connie Brand, Ruth A. Mulnard, Gaby Thai, Catherine McAdams-Ortiz, Kyle Womack, Dana Mathews, Mary Quiceno, Ramon Diaz-Arrastia, Richard King, Myron Weiner, Kristen Martin-Cook, Michael DeVous, Allan I. Levey, James J. Lah, Janet S. Cellar, Jeffrey M. Burns, Heather S. Anderson, Russell H. Swerdlow, Liana Apostolova, Kathleen Tingus, Ellen Woo, Daniel H. S. Silverman, Po H. Lu, George Bartzokis, Neill R. Graff-Radford, Francine Parfitt, Tracy Kendall, Heather Johnson, Martin R. Farlow, Ann Marie Hake, Brandy R. Matthews, Scott Herring, Cynthia Hunt, Christopher H. van Dyck, Richard E. Carson, Martha G. MacAvoy, Howard Chertkow, Howard Bergman, Chris Hosein, Ging-Yuek Robin Hsiung, Howard Feldman, Benita Mudge, Michele Assaly, Charles Bernick, Donna Munic, Andrew Kertesz, John Rogers, Dick Trost, Diana Kerwin, Kristine Lipowski, Chuang-Kuo Wu, Nancy Johnson, Carl Sadowsky, Walter Martinez, Teresa Villena, Raymond Scott Turner, Kathleen Johnson, Brigid Reynolds, Reisa A. Sperling, Keith A. Johnson, Gad Marshall, Meghan Frey, Barton Lane, Allyson Rosen, Jared Tinklenberg, Marwan N. Sabbagh, Christine M. Belden, Sandra A. Jacobson, Sherye A. Sirrel, Neil Kowall, Ronald Killiany, Andrew E. Budson, Alexander Norbash, Patricia Lynn Johnson, Joanne Allard, Alan Lerner, Paula Ogrocki, Leon Hudson, Evan Fletcher, Owen Carmichae, John Olichney, Charles DeCarli, Smita Kittur, Michael Borrie, T.-Y. Lee, Rob Bartha, Sterling Johnson, Sanjay Asthana, Cynthia M. Carlsson, Adrian Preda, Dana Nguyen, Pierre Tariot, Stephanie Reeder, Vernice Bates, Horacio Capote, Michelle Rainka, Douglas W. Scharre, Maria Kataki, Anahita Adeli, Earl A. Zimmerman, Dzintra Celmins, Alice D. Brown, Godfrey D. Pearlson, Karen Blank, Karen Anderson, Robert B. Santulli, Tamar J. Kitzmiller, Eben S. Schwartz, Kaycee M. Sink, Jeff D. Williamson, Pradeep Garg, Franklin Watkins, Brian R. Ott, Henry Querfurth, Geoffrey Tremont, Stephen Salloway, Paul Malloy, Stephen Correia, Howard J. Rosen, Bruce L. Miller, Jacobo Mintzer, Kenneth Spicer, David Bachman, Stephen Pasternak, Irina Rachinsky, Dick Drost, Nunzio Pomara, Raymundo Hernando, Antero Sarrael, Susan K. Schultz, Laura L. Boles Ponto, Hyungsub Shim, Karen Elizabeth Smith, Norman Relkin, Gloria Chaing, Lisa Raudin, Amanda Smith, Kristin Fargher, Balebail Ashok Raj, Thomas Neylan, Jordan Grafman, Melissa Davis, Rosemary Morrison, Jacqueline Hayes, Shannon Finley, Karl Friedl, Debra Fleischman, Konstantinos Arfanakis, Olga James, Dino Massoglia, JJay Fruehling, Sandra Harding, Elaine R. Peskind, Eric C. Petrie, Gail Li, Jerome A. Yesavage, Joy L. Taylor, Ansgar J. Furst

**Affiliations:** 1 School of Communication and Information Engineering, Shanghai University, Shanghai, 200444 China; 2 Department of Nuclear Medicine, the Second Hospital of Zhejiang University School of Medicine, Hangzhou, 310009 Zhejiang China; 3 Institute of Biomedical Engineering, School of Life Sciences, Shanghai University, Shanghai, 200444 China; 4 Department of Neurology, Xuanwu Hospital of Capital Medical University, Beijing, 100053 China; 5 Department of Neurology, Shanghai Pudong Hospital, Fudan University Pudong Medical Center, 2800 Gongwei Road, Shanghai, 201399 Pudong China

**Keywords:** Alzheimer’s disease, Imaging biomarkers, Functional connectivity, Graph neural network, Multi-site

## Abstract

**Background:**

Functional connectivity (FC) biomarkers play a crucial role in the early diagnosis and mechanistic study of Alzheimer’s disease (AD). However, the identification of effective FC biomarkers remains challenging. In this study, we introduce a novel approach, the spatiotemporal graph convolutional network (ST-GCN) combined with the gradient-based class activation mapping (Grad-CAM) model (STGC-GCAM), to effectively identify FC biomarkers for AD.

**Methods:**

This multi-center cross-racial retrospective study involved 2,272 participants, including 1,105 cognitively normal (CN) subjects, 790 mild cognitive impairment (MCI) individuals, and 377 AD patients. All participants underwent functional magnetic resonance imaging (fMRI) and T1-weighted MRI scans. In this study, firstly, we optimized the STGC-GCAM model to enhance classification accuracy. Secondly, we identified novel AD-associated biomarkers using the optimized model. Thirdly, we validated the imaging biomarkers using Kaplan–Meier analysis. Lastly, we performed correlation analysis and causal mediation analysis to confirm the physiological significance of the identified biomarkers.

**Results:**

The STGC-GCAM model demonstrated great classification performance (The average area under the curve (AUC) values for different categories were: CN vs MCI = 0.98, CN vs AD = 0.95, MCI vs AD = 0.96, stable MCI vs progressive MCI = 0.79). Notably, the model identified specific brain regions, including the sensorimotor network (SMN), visual network (VN), and default mode network (DMN), as key differentiators between patients and CN individuals. These brain regions exhibited significant associations with the severity of cognitive impairment (*p* < 0.05). Moreover, the topological features of important brain regions demonstrated excellent predictive capability for the conversion from MCI to AD (Hazard ratio = 3.885, *p* < 0.001). Additionally, our findings revealed that the topological features of these brain regions mediated the impact of amyloid beta (Aβ) deposition (bootstrapped average causal mediation effect: β = -0.01 [-0.025, 0.00], *p* < 0.001) and brain glucose metabolism (bootstrapped average causal mediation effect: β = -0.02 [-0.04, -0.001], *p* < 0.001) on cognitive status.

**Conclusions:**

This study presents the STGC-GCAM framework, which identifies FC biomarkers using a large multi-site fMRI dataset.

**Supplementary Information:**

The online version contains supplementary material available at 10.1186/s13195-024-01425-8.

## Background

Alzheimer’s disease (AD) is a prevalent neurodegenerative disorder responsible for 60–80% of dementia cases [1]. To achieve early clinical diagnosis and quantify the stage of the disease, it is crucial to identify potential biomarkers or neuroimaging indicators [2]. Ongoing research focuses on utilizing various neuroimaging techniques, including magnetic resonance imaging (MRI) [3–5], functional magnetic resonance imaging (fMRI) [6], positron emission tomography (PET) [7], and computed tomography (CT) [8], to detect AD-related biomarkers and enable early diagnosis.

Resting-state fMRI (rs-fMRI) is instrumental in capturing brain activity by detecting changes in blood oxygen levels. It has been extensively utilized in identifying imaging markers related to AD and has revealed AD to be a "functional disconnection syndrome" [9]. Research reports have consistently demonstrated reduced FC in specific brain regions, including the posterior cingulate cortex, medial prefrontal cortex, inferior parietal cortex, inferior temporal cortex, and hippocampus, in patients with AD [10, 11]. However, conventional brain functional network analysis methods typically focus on group comparisons between patients and healthy controls [12]. Given the presence of individual differences in AD pathology arising from factors like age, sex, genetics, and ethnicity, the challenge lies in establishing objective and stable FC biomarkers that account for the heterogeneity in brain functional network patterns. Additionally, the inclusion of large sample sizes is vital to ensure the overall representativeness of models and provide reliable information about the underlying biological basis. Consequently, there is a growing emphasis on the integration of multi-factor analysis using combined datasets from large samples [13].

Deep learning methods have gained prominence in medical imaging analysis as they can automatically capture high-dimensional features [14–16]. For instance, convolutional neural networks (CNNs) have demonstrated significant efficacy across diverse tasks, notably in applications like AD classification [17]. Nonetheless, employing CNN models to model fMRI data presents specific challenges. These include the constraint of spatial features to a limited neighborhood in Euclidean space, particularly noticeable in shallow CNN models. Moreover, brain region distributions are irregular, posing challenges in capturing intricate connectivity features [18]. Therefore, fMRI exhibits intricate spatiotemporal interaction patterns that cannot be adequately captured through CNN [19]. In the context of brain connectivity patterns, graph convolutional neural networks (GCNs) have been proposed for processing brain connectivity networks due to their structural compatibility [20]. GCN analyzes the graph structure and node characteristics by performing graph convolutions, effectively capturing more discriminative features [21]. Currently, numerous researchers have combined GCN and fMRI for AD diagnosis. For example, Mei et al. [22] proposed a hierarchical GCN model for MCI diagnosis. Addressing the integration of spatiotemporal information within brain networks, Wang et al. [23] sampled fMRI in adjacent space and time to learn spatial–temporal features. In [24, 25], spatial and temporal features were derived by employing GCNs and Recurrent Neural Networks (RNNs), respectively. However, existing GCN models for AD diagnosis exhibit two notable limitations: (1) The current GCN models fail to seamlessly integrate spatiotemporal information, resulting in a compromise to the continuity of fMRI data; (2) These models cannot precisely identify brain regions as biomarkers in disease classification.

Therefore, we propose a novel model called spatiotemporal graph convolution combined with gradient-based class activation mapping (STGC-GCAM) to identify imaging biomarkers for AD and its stages. In this model, spatial–temporal GCN (ST-GCN) obtains disease diagnostic features by integrating spatiotemporal information from the brain’s functional connection network [26]. Specifically, spatial convolutions operate on the spatial dimension, while temporal convolutions operate on the temporal dimension [27]. The gradient-based class activation mapping (Grad-CAM) technique assigns weights to nodes based on gradient information of specific classes, thereby highlighting the contribution of important nodes.

In this study, our objective was to identify novel FC biomarkers for AD using the STGC-GCAM model. We employed a large multi-site dataset comprising 2272 participants. The first hypothesis posits that the STGC-GCAM model can detect abnormal brain regions at the network level using fMRI data. The second hypothesis suggests that the ST-GCN approach can reveal network topological defects associated with clinical variables. To validate these hypotheses, the STGC-GCAM model was trained on brain FC networks to characterize AD patients and their stages (mild cognitive impairment (MCI), stable MCI (sMCI), progressive MCI (pMCI)). Additionally, the study identified significant brain regions that contribute to accurate classification.

## Methods

### Participants

Our study utilized three cohorts: cohort I from the Alzheimer’s Disease Neuroimaging Initiative (ADNI) database (http://adni.loni.usc.edu/), cohort II from the Sino Longitudinal Study on Cognitive Decline (SILCODE) at Xuanwu Hospital of Capital Medical University in Beijing, China, and cohort III from Tongji Hospital in Shanghai, China. The data from the three cohorts were divided into six sites based on the length of the fMRI time series. The study included 1,105 cognitively normal (CN) participants, 790 participants with MCI, and 377 participants with AD. Comprehensive data, including demographic information (gender, age, education level), neuropsychological assessments (mini-mental state examination (MMSE), the sum of boxes of clinical dementia rating scale (CDR-SB)), cerebrospinal fluid tau/phosphorylated tau (CSF TAU/PTAU), fMRI, and T1-weighted MRI data, were collected for all participants. Specifically, fMRI is utilized for model training, T1 data is employed for registration during fMRI preprocessing, and additional data modalities are used for result validation and analysis. Additionally, for cohorts I and II, we investigated the conversion of MCI participants to AD within a 3-year timeframe to validate the model’s effectiveness in different tasks. Further details are provided in Table 1.

**Table 1:** Demographic and clinical characteristics of included multi-site participants

**Cohort**	**ADNI**						Xuanwu						**Tongji**					
**Site**	**site1**			**site2**			**site3**			**site4**			**site5**			**site6**		
**Group**	CN *n* = 139	MCI *n* = 244	AD *n* = 132	CN *n* = 440	MCI *n* = 232	AD *n* = 90	CN *n* = 148	MCI *n* = 121	AD *n* = 38	CN *n* = 241	MCI *n* = 46	AD *n *= 18	CN *n* = 71	MCI *n* = 85	AD *n* = 57	CN *n* = 66	MCI *n* = 62	AD *n *= 42
**Gender** (M/F)	58 /81	138 /106^a^	69 /63	176 /264	127 /105^a^	58 /32^b^	71 /77	50 /71^a^	15 /23^b^	101 /140	22 /24	7 /11	40 /31	38 /47	36 /21^b^	31 /35	28 /34	19 /23
**Age** (mean ± SD)	75.4 ± 6.1	73.0 ± 8.1	74.5 ± 7.2	71.0 ± 6.3	76.0 ± 8.3^a^	77.4 ± 7.9^b^	64.0 ± 12.5	69.0 ± 9.8^a^	73.0 ± 8.8^b^	66.2 ± 5.2	68.3 ± 9.6^a^	70.4 ± 11.3^b^	71.0 ± 8.0	71.6 ± 8.1	73.7 ± 8.3	68.0 ± 6.7	73.1 ± 6.1^a^	74.4 ± 8.8^b^
**Edu** (mean ± SD)	16.7 ± 2.1	16.1 ± 2.6^a^	15.6 ± 2.8^b^	16.3 ± 2.1	15.7 ± 2.8^a^	15.6 ± 2.6^b^	12.0 ± 8.3	10.0 ± 4.5^a^	9.0 ± 5.0^b^	12.4 ± 3.2	11.5 ± 4.2	13.8 ± 2.9	12.0 ± 3.7	10.9 ± 4.3^a^	8.3 ± 5.4^b^	12.4 ± 3.4	9.0 ± 4.0^a^	8.7 ± 5.3^b^
**sMCI** **/pMCI** (conversion time)	-	164/80 (21.0 ± 12.0)	-	-	214/17 (21.4 ± 6.3)	-	-	63/58 (22.0 ± 15.0)	-	-	35/11 (17.3 ± 4.3)	-	-	-	-	-	-	-
**APOE**ε4 (-/ +)	83 /56	147 /97^a^	16 /116^b^	261 /142	136 /78	36 /44^b^	67 /28	51 /57^a^	18 /13	193 /47	25 /19^a^	8 /9^b^	-	-	-	-	-	-
**Aβ** (-/ +)	31 /13	34/ 42^a^	4/ 39^b^	191 /60	57 /32^a^	7 /40^b^	23 /0	0 /1	0 /4	70 /13	4 /5	2 /2	-	-	-	-	-	-
**MMSE** (mean ± SD)	29.0 ± 1.4	27.8 ± 2.0^a^	22.0 ± 3.6^b^	28.9 ± 1.6	27.6 ± 2.0^a^	21.4 ± 4.2^b^	28.0 ± 2.1	24.0 ± 3.6^a^	18.0 ± 3.8^b^	28.8 ± 1.3	25.6 ± 2.9^a^	16.8 ± 71^b^	27.0 ± 2.2	23.8 ± 3.3^a^	14.7 ± 6.2^b^	27.2 ± 2.1	23.9 ± 2.8^a^	15.1 ± 5.3^b^
**CDR-SB** (mean ± SD)	0.1 ± 0.2	1.5 ± 1.1^a^	4.9 ± 1.9^b^	0.08 ± 0.3	1.5 ± 1.1^a^	6.0 ± 3.0^b^	-	-	-	-	-	-	-	-	-	-	-	-

Age, Education, and MMSE data were given as mean standard ± SD. ^a^ *p* < 0.05, comparison between CN and MCI. ^b^ *p* < 0.05, comparison between CN and AD. *Abbreviations*: *CDR-SB* The sum of boxes of clinical dementia rating scale, *CN* Cognitively normal, *Edu* Education, *MMSE* Mini-Mental State Examination, *MCI* Mild cognitive impairment, *SD* Standard deviation, *pMCI* Progressive MCI, *sMCI* Stable MCI

Cohort I consisted of 579 CN, 222 AD, and 476 MCI participants, including 378 participants with sMCI and 97 participants with pMCI. APOE ε4 and amyloid beta (Aβ) information were also collected from participants in cohort I, following the diagnostic criteria described in the ADNI manual (http://www.adni-info.org). The inclusion criteria for MCI patients from the ADNI database have been previously reported [28]. Please refer to the supplementary material (Participants: Cohort I) for more details.

The SILCODE project aims to diagnose subject cognitive decline (SCD) and MCI in the early stages of AD using multimodal data, including clinical information, neuropsychological assessment, biological markers, and neuroimaging [29]. Cohort II consisted of 389 CN individuals, 56 AD patients, and 167 MCI patients, including 98 participants with sMCI and 69 participants with pMCI. APOE ε4 and Aβ information was also collected from the participants. The inclusion criteria for cohort II data were previously reported [30], and the entry criteria for CN individuals [29] and the diagnostic criteria for AD dementia [31] and MCI [32] were in accordance with established guidelines. Please refer to the supplementary material (Participants: Cohort II) for more details.

Cohort III, obtained from Tongji Hospital, consisted of 143 CN individuals, 99 AD patients, and 147 MCI patients. However, data in cohort III did not include information on APOE ε4 and Aβ, and there was no available follow-up information. Diagnoses for all participants were made by experienced clinical physicians, with MCI primarily diagnosed based on neuropsychological testing [33] and AD diagnosed according to the criteria established by the National Institute on Aging (NIA) [34].

The fMRI data exclusion criteria and inclusion flowcharts are provided in the supplementary material (Figure S2-S4). Ethical approval for all studies was obtained from the respective institutional review committees, and written informed consent was obtained from each participant.

### Neuroimaging acquisition and preprocessing

Participants in this study underwent fMRI and T1-MRI scans. Detailed information regarding the scanning equipment and acquisition parameters for each site can be found in the supplementary material (Table S3). A standardized image preprocessing protocol was applied using the Data Processing Assistant for Resting-State fMRI (DPARSF) (http://restfmri.net/forum/DPARSF) [35, 36]. The first ten volumes of the fMRI data were discarded to achieve magnetization balance, and the remaining volumes underwent correction for slicing time and realignment to the first volume for head motion. Participants exceeding the thresholds of 3.0 mm translation and 1.0° rotation were excluded (see Figure S2-S4). Registration was performed by aligning the functional image to the T1 images. Subsequently, normalization to Montreal Neurological Institute (MNI) space was carried out with a spatial resolution of 3 mm × 3 mm × 3 mm. Baseline drift removal was performed, and the fMRI images were corrected for 24 head movement parameters to mitigate nuisance signals and account for head motion effects. Furthermore, regression of white matter and cerebrospinal fluid signals was conducted to attenuate respiratory and cardiac effects. The resulting images were temporally filtered (0.01–0.08 Hz) and spatially smoothed (6 mm FWHM Gaussian kernel). In the multi-site context, various factors including age, gender, different scanners, acquisition parameters, and inter-site confounding effects can influence the analysis. Despite employing the same image processing method, eliminating cross-site nuisance variables remains challenging and can significantly impact the performance of the model [13]. To address this issue, we adopted ComBat harmonization, a method commonly used in genomic studies [37].

### Network Architectures of STGC-GCAM

**Fig. 1:**
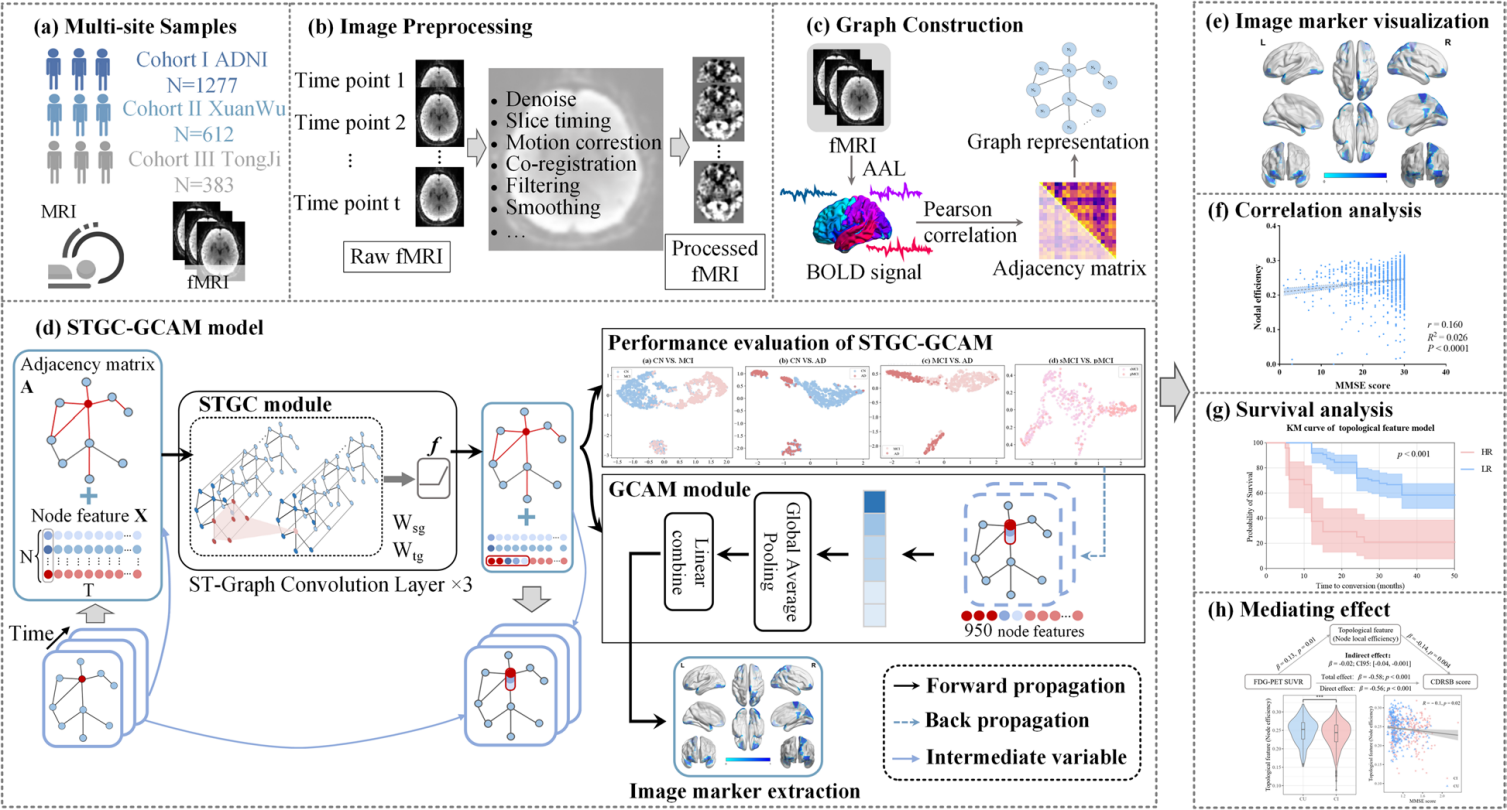
Comprehensive workflow of the study. **a** Data collection from three centers: ADNI, Xuanwu Hospital, and Tongji Hospital. **b** fMRI preprocessing flow. **c** Graph representation construction based on fMRI. **d** Architecture of the STGC-GCAM model. **e** Visualization of imaging biomarkers using BrainNet Viewer. **f** Correlation analysis between topological features of disease-related brain regions and clinical indicators. **g** Survival analysis of MCI patients using imaging markers. **h** Investigation of the mediating effects of brain region topology characteristics on cognitive disorders caused by Aβ, tau, and neural variants

We employed ST-GCN to analyze fMRI data, capturing both temporal and spatial information. This approach allowed for the extraction of detailed features to aid in early diagnosis of AD. To identify relevant brain regions associated with AD and its stages, we introduced the STGC-GCAM model. Figure 1 illustrates the study’s workflow, highlighting the architecture of the STGC-GCAM model designed specifically for processing fMRI images (Fig. 1(d), and Figure S1).

#### Brain network construction

To construct the individual dynamic FC brain network from fMRI data, we created a weighted undirected spatiotemporal graph $G\;{ = }\left\{ {{\mathbf{V}},\;{\mathbf{E}},\;{\mathbf{A}}} \right\}$. The graph G consists of nodes ${|}{\mathbf{V}}{|}\;{\text{ = N}}$ and edges **E** containing spatiotemporal information, represented by the weighted adjacency matrix ${\mathbf{A}} \in {\mathbb{R}}^{{{\text{N}} \times {\text{N}}}}$. N is the number of nodes, which in this study is also the number of brain regions. In this study, we used the Anatomical Automatic Labeling (AAL) atlas [38] to define 90 brain nodes and extracted the average blood oxygen level-dependent (BOLD) time series for each node. The adjacency matrix **A** was determined by calculating the Pearson correlation coefficient between the BOLD signals of different nodes [39]. Figure 1(c) illustrates the process of constructing the brain network using fMRI data. These constructed spatiotemporal graphs served as inputs for the STGC-GCAM model.

#### Spatiotemporal graph convolution

The STGC-GCAM model incorporates ST-GCN to extract valuable spatiotemporal features from fMRI data to aid in early AD diagnosis. The model takes node features **X** and the adjacency matrix **A** as inputs. Where the node feature ${\text{X}} \in {\mathbb{R}}^{{T \times {\text{N}}}}$ is represented as the BOLD of the node extracted from fMRI, T = 950 represents the number of time points of the node. The spatiotemporal convolution is approximated by decomposing it into spectral-domain spatial graph convolution and temporal convolution, as described in the reference [40]. Equations (1–2) represent the spatial graph convolution layer and temporal convolution, respectively.

1 $$\mathbf{Z}^{j^\prime}_\text{sg}=Relu\left(\mathbf{D}^{-\frac{1}{2}}(\mathbf{A}+\mathbf{I})\mathbf{D}^{-\frac{1}{2}}\mathbf{Z}^\text{j}_\text{sg}\mathbf{W}_\text{sg}\right)$$

2 $$\mathbf Z_{\mathrm{tg}}^{\mathrm{ij'}}\;=Relu\;\left(\mathbf Z_{\mathrm{tg}}^{\mathrm{ij}}\;\otimes\;{\mathbf W}_{\mathrm{tg}}\right)$$

where, $\mathbf{Z}^{\text{j}^\prime}_\text{sg}=\in\mathbb{R}^{\text{N}\times\text{M}}$ represents the M-channel output features of the N nodes at the j-th layer of spatial convolution, $Relu{(} \bullet {)}$ is the rectifiers linear unit, **D** is a diagonal matrix, **I** represents the identity matrix, ${\mathbf{Z}}_{{{\text{sg}}}}^{{\text{j}}} \in {\mathbb{R}}^{{{\text{N}} \times {\text{C}}}}$ represents the C-channel output features, ${\mathbf{W}}_{{{\text{sg}}}} \in {\mathbb{R}}^{{{\text{C}} \times {\text{M}}}}$ represents spatial graph convolution kernel. $\mathbf{Z}^{\text{ij}\prime}_\text{tg}=\in\mathbb{R}^{\text{M}\times\Gamma}$ represents the output features of node i at the j-th layer temporal convolution, ${\mathbf{Z}}_{{{\text{tg}}}}^{{{\text{ij}}}} \in {\mathbb{R}}^{{{\text{M}} \times {\text{T}}}}$ represents the input feature of node i, ${\mathbf{W}}_{{{\text{tg}}}} \in {\mathbb{R}}^{{{\text{M}} \times {\Gamma }}}$ represents the temporal convolution kernel, Γ represents the size of the temporal convolution kernel. $\otimes$ represents convolution in the time domain.

In this study, we introduce a spatiotemporal graph convolutional unit, which combines spatial and temporal convolutions. Our model consists of three such units, with output channel numbers of 128, 64, and 32 for each layer. Following the convolutions, global average pooling and fully connected layers are utilized to obtain predicted probabilities for each class. For a more detailed introduction, please refer to the supplementary materials (Training strategy).

#### Imaging biomarker identification

We employed Grad-CAM to estimate the contribution of each brain region to classification. Grad-CAM is an improvement upon the class activation mapping (CAM) method [41] proposed by Zhou et al. [42], enhancing its application and simplifying the model architecture. Incorporating Grad-CAM into ST-GCN has been suggested in a recent study [43]. The spatiotemporal graph convolutional layer in ST-GCN captures local temporal and spatial information, reflecting the spatiotemporal importance of nodes. In Grad-CAM, the gradient information from the last spatiotemporal graph convolutional unit is used to compute node importance for decision-making. Detailed calculation procedures for Grad-CAM (Figure S1). The algorithm flow of STGC-GCAM is described in Table S1, and parameter settings for the STGC-GCAM model can be found in the supplementary material (Table S2).

### Training of STGC-GCAM model and comparison models

The performance evaluation of the STGC-GCAM model encompassed various tasks, including additional inter-group analyses involving CN and MCI, CN and AD, MCI and AD, as well as sMCI and pMCI, in addition to the primary analysis comparing CN and MCI patients. Hyperparameter tuning for the model was conducted through fine-tuning using a grid search approach. In order to achieve better results, we trained the models separately on different classification tasks (CN vs. MCI, CN vs. AD, MCI vs. AD, sMCI vs. pMCI). Optimal hyperparameters were determined via this method on the training set. A fivefold cross-validation strategy was adopted for both parameter tuning on the training set and for verifying the performance of the model on the test set. In this procedure, the entire dataset was initially partitioned into training sets (80%) and a test set (20%). The training set and test set were further subdivided into 5 subsets of roughly equal size. Since this research involves multi-center data, two points need to be ensured when dividing the data set: (1) the proportion of positive and negative samples in the training set and the test set is consistent; (2) the proportion of each center in the training set and test set is consistent. Within the training set, one of the subsets was designated as the validation set, while the other four were employed for hyperparameter tuning. On the test set, the best model was identified using the 4 subsets and saved, with the final results obtained from the remaining subset. The classification results were averaged over all iterations to yield a comprehensive assessment of model performance across different tasks, quantified by metrics such as accuracy (ACC), sensitivity (SEN), specificity (SPE), and area under the curve (AUC).

To visualize the distribution of image features within a classification network, we employed t-distributed stochastic neighbor embedding (t-SNE) [44]. Secondly, we utilized Grad-CAM to analyze the important brain regions of individual subjects and visualized the top 10 important brain regions with the highest frequency using BrainNet Viewer [45]. Thirdly, we evaluated the classification performance of the model using data from a single site and compared its variability to that of the model trained on multicenter data. Fourthly, we compared the performance of the classification network with other classifiers, including GCN [13], which utilizes spectral graph convolution to operate on irregular graph data instead of Euclidean data. The input to GCN is the same as the input to ST-GCN. We also included BrainCNN [46], a CNN framework designed for predicting clinical neurodevelopmental outcomes from brain networks, whose input is the adjacency matrix. In addition, we evaluated the performance of traditional machine learning classifiers, namely support vector machine (SVM), multilayer perceptron (MLP), logistic regression (LR), and random forest (RF), in the classification task. The input of traditional machine learning methods only includes node features.

The Adam optimizer [47], implementing adaptive learning rates, was employed to update model weights. Additionally, a stepwise learning rate decay strategy was adopted in case validation accuracy plateaued, setting the minimum learning rate to 0.001 (0.01, 0.001). Evaluation on the validation dataset occurred after each training epoch, saving the model parameters only upon achieving improved validation accuracy. L2 regularization was integrated during training to mitigate overfitting. The ultimate performance metric was determined by selecting the highest accuracy achieved across distinct L2 regularization values (0.1, 0.01, and 0.001). We implement the proposed STGC-GCAM based on Pytorch 1.11 (Python 3.8) [48] and the model was trained using a single GPU (NVIDIA TITAN Xp with 12GB memory).

### Imaging marker identification and validation

Each brain region identified using the Automated Anatomical Labeling (AAL) atlas corresponds to a specific resting state network (RSN) [49]. The RSN extracted from the network templates consist of the default mode network (DMN), attention network (AN), sensorimotor network (SMN), subcortical network, and visual network (VN) (Supplementary Material Table S4). To evaluate the pathological interpretability of the STG-GCAM model, we analyzed the distribution of the identified important brain regions within the RSN. Additionally, we examined the correlation between the topological characteristics of these brain regions and disease severity using the Spearman correlation.

We employed a multifactor Cox proportional hazards regression model to evaluate the predictive value of important brain region features as biomarkers for predicting progression from MCI to AD. The survival time of individuals with pMCI was defined as the duration between the baseline fMRI imaging time and the initial AD diagnosis or the last follow-up time for MCI subjects. The STGC-GCAM was used to identify disease-affected brain regions, from which features such as topological features, regional homogeneity (ReHo), and amplitude of low-frequency fluctuation (ALFF) were extracted as predictors. Three prediction models were constructed in this study: one based on ALFF, another based on ReHo, and the third on topological features. The topological feature-based model included nodal efficiency as a measure of information transfer efficiency between nodes in the network, encompassing both global (nodal efficiency, NE) and local (nodal local efficiency, NLE) measures [50]. Risk values derived from the models were used to stratify the sample into low-risk and high-risk groups. Survival analysis using Kaplan–Meier (KM) was conducted to compare the risk groups.

### Causal mediation analyses

We performed mediation regression analysis on the site1 and site2 datasets to investigate the extent to which the effects of biomarkers of significant brain regions on cognitive status are mediated by the topological features. Four models were constructed using global AV45-PET SUVR, CSF TAU/PTAU, and FDG-PET SUVR as predictor variables, estimating the direct and indirect (mediation) effects of the topological features of important brain regions on neurocognitive scale scores. According to the information provided by ADNI, the AV45-PET SUVR for the entire brain can be calculated by using the cerebellum as a reference region. FDG-PET SUVR is the average FDG-PET of angular, temporal, and posterior cingulate. See Jagust LABS PDF on LONI for details. We adjusted for potential mediator-outcome confounders, including age, sex, education years, and APOE ε4. To address the comparability of the data metrics, we standardized each variable in the analysis. All mediation analyses were conducted using the PROCESS module in SPSS, and the results were reported as unstandardized effect sizes [51]. We used bootstrap procedures with 5000 replications to compute a 95% bias-corrected bootstrap confidence interval (95% BCCI) for the indirect effects.

### Statistical analysis

To investigate differences between groups in the variables listed in Table 1, we employed statistical tests appropriate for the data type. Specifically, the chi-square test was used for categorical variables, and the independent two-sample t-test was used for continuous variables. In the analysis of multi-site data, Spearman correlation was utilized to examine the relationship between important brain region features and neuropsychological assessments in both the disease and normal groups. Hazard ratios and associated 95% confidence intervals (CI) were estimated using the Cox proportional hazards regression model. The log-rank test was used to assess between-group differences in KM overall survival for MCI patients (high-risk and low-risk groups). All the above statistical analyses were performed using SPSS 25.0, and *p*-values less than 0.05 (two-tailed) were considered statistically significant.

## Results

### Demographic information and neuropsychological assessments

Table 1 shows the demographic and clinical information of all participants. In site 1, significant differences (*p* < 0.01) were observed between the CN and MCI groups and between the CN and AD groups in terms of education level, APOE ε4, Aβ, MMSE, and CDR-SB, while there were no differences in gender and age. In site 2, there were significant differences (*p* < 0.01) between the CN and MCI groups, as well as the CN and AD groups, in terms of gender, age, education level, APOE ε4, Aβ, MMSE, and CDR-SB. In site 3, only the CN and AD groups showed no significant differences in APOE ε4, while significant differences were observed in other aspects between the CN and MCI groups, as well as between the CN and AD groups (*p* < 0.01). In site 4, there were significant differences (*p* < 0.05) between the CN and MCI groups as well as the CN and AD groups in terms of age, MMSE, and APOE ε4, with no significant differences in gender and education level. In site 5, there were significant differences (*p* < 0.05) between the CN and MCI groups in terms of education level and MMSE, with no significant differences in age and gender; and significant differences (*p* < 0.05) between the CN and AD groups in terms of gender, education level, and MMSE, with no significant differences in age.

### Evaluation of the performance of STGC-GCAM models and comparison models

We employed a fivefold cross-validation approach on ST-GCN to integrate data from multiple sites for the diagnosis of AD and its stages. the ST-GCN achieved a classification accuracy of 0.93 ± 0.001 (AUC = 0.98 ± 0.001) for CN and MCI. The accuracy for differentiating AD from CN was 0.90 ± 0.002 (AUC = 0.95 ± 0.002), while for differentiating AD from MCI patients, it was 0.92 ± 0.002 (AUC = 0.96 ± 0.002). In addition, the accuracy for distinguishing between sMCI and pMCI was 0.85 ± 0.002 (AUC = 0.79 ± 0.002) (Table 2). The scatterplot in Figure S5 shows the distribution of image features in a binary classification network.

**Table 2:** STGC-GCAM model performance under different classification tasks. Values are reported in terms of mean ± standard deviation

CN vs. MCI					CN vs. AD				MCI vs. AD				sMCI vs. pMCI			
Model	**ACC**	**SEN**	**SPE**	**AUC**	**ACC**	**SEN**	**SPE**	**AUC**	**ACC**	**SEN**	**SPE**	**AUC**	**ACC**	**SEN**	**SPE**	**AUC**
SVM	0.64 ± 0.003	0.52 ± 0.010	0.73 ± 0.010	0.62 ± 0.003	0.59 ± 0.004	0.32 ± 0.010	0.68 ± 0.004	0.50 ± 0.010	0.64 ± 0.004	0.21 ± 0.010	0.84 ± 0.010	0.53 ± 0.004	0.65 ± 0.010	0.30 ± 0.010	0.77 ± 0.010	0.53 ± 0.010
MLP	0.64 ± 0.004	0.53 ± 0.020	0.71 ± 0.010	0.62 ± 0.010	0.75 ± 0.003	0.22 ± 0.010	0.93 ± 0.010	0.58 ± 0.004	0.62 ± 0.004	0.14 ± 0.010	0.87 ± 0.010	0.50 ± 0.003	0.71 ± 0.010	0.21 ± 0.010	0.88 ± 0.010	0.54 ± 0.010
RF	0.63 ± 0.004	0.45 ± 0.004	0.76 ± 0.010	0.61 ± 0.004	0.74 ± 0.003	0.12 ± 0.010	0.96 ± 0.003	0.54 ± 0.004	0.69 ± 0.002	0.12 ± 0.010	0.94 ± 0.004	0.54 ± 0.002	0.64 ± 0.010	0.32 ± 0.020	0.74 ± 0.010	0.53 ± 0.010
LR	0.64 ± 0.003	0.48 ± 0.010	0.76 ± 0.010	0.62 ± 0.004	0.75 ± 0.003	0.21 ± 0.010	0.93 ± 0.002	0.57 ± 0.004	0.65 ± 0.010	0.11 ± 0.010	0.90 ± 0.010	0.51 ± 0.010	0.73 ± 0.010	0.14 ± 0.010	0.93 ± 0.010	0.53 ± 0.010
Brain CNN	0.72 ± 0.010	0.77 ± 0.010	0.65 ± 0.040	0.76 ± 0.010	0.76 ± 0.010	0.72 ± 0.010	0.78 ± 0.010	0.81 ± 0.010	0.64 ± 0.010	0.33 ± 0.040	0.79 ± 0.030	0.60 ± 0.004	0.77 ± 0.004	0.62 ± 0.010	0.82 ± 0.010	0.78 ± 0.010
GCN	0.83 ± 0.003	0.79 ± 0.020	0.85 ± 0.010	0.86 ± 0.010	0.81 ± 0.010	0.53 ± 0.050	0.91 ± 0.020	0.78 ± 0.030	0.68 ± 0.002	0.49 ± 0.010	0.77 ± 0.010	0.62 ± 0.010	0.79 ± 0.010	0.61 ± 0.010	0.85 ± 0.010	0.74 ± 0.010
STGC-GCAM	**0.93 ± 0.001**	**0.87 ± 0.010**	**0.97 ± 0.003**	**0.98 ± 0.001**	**0.90 ± 0.002**	**0.99 ± 0.002**	**0.87 ± 0.002**	**0.95 ± 0.002**	**0.92 ± 0.002**	**0.97 ± 0.003**	**0.89 ± 0.002**	**0.96 ± 0.002**	**0.85 ± 0.002**	**0.67 ± 0.009**	**0.92 ± 0.006**	**0.79 ± 0.002**

*Abbreviations*: *ACC* Accuracy, *AUC* Area under receiver operating characteristic curve, *SEN* Specificity, *SPE* Specificity

In single-center-based classification tasks, we found that the classification accuracy of CN and MCI was between 0.62 to 0.84, the classification accuracy of CN and AD was between 0.66 to 0.93, the classification accuracy of MCI and AD was between 0.61 to 0.80, and the classification accuracy of sMCI and pMCI was between 0.66 to 0.94 (Table S5). In addition, STGC-GCAM demonstrates superior classification accuracy compared to other commonly used machine learning algorithms (SVM, RF, MLP, and LR), as well as BrainCNN and GCN, as shown in Table 2.

### Imaging biomarkers identification

**Fig. 2:**
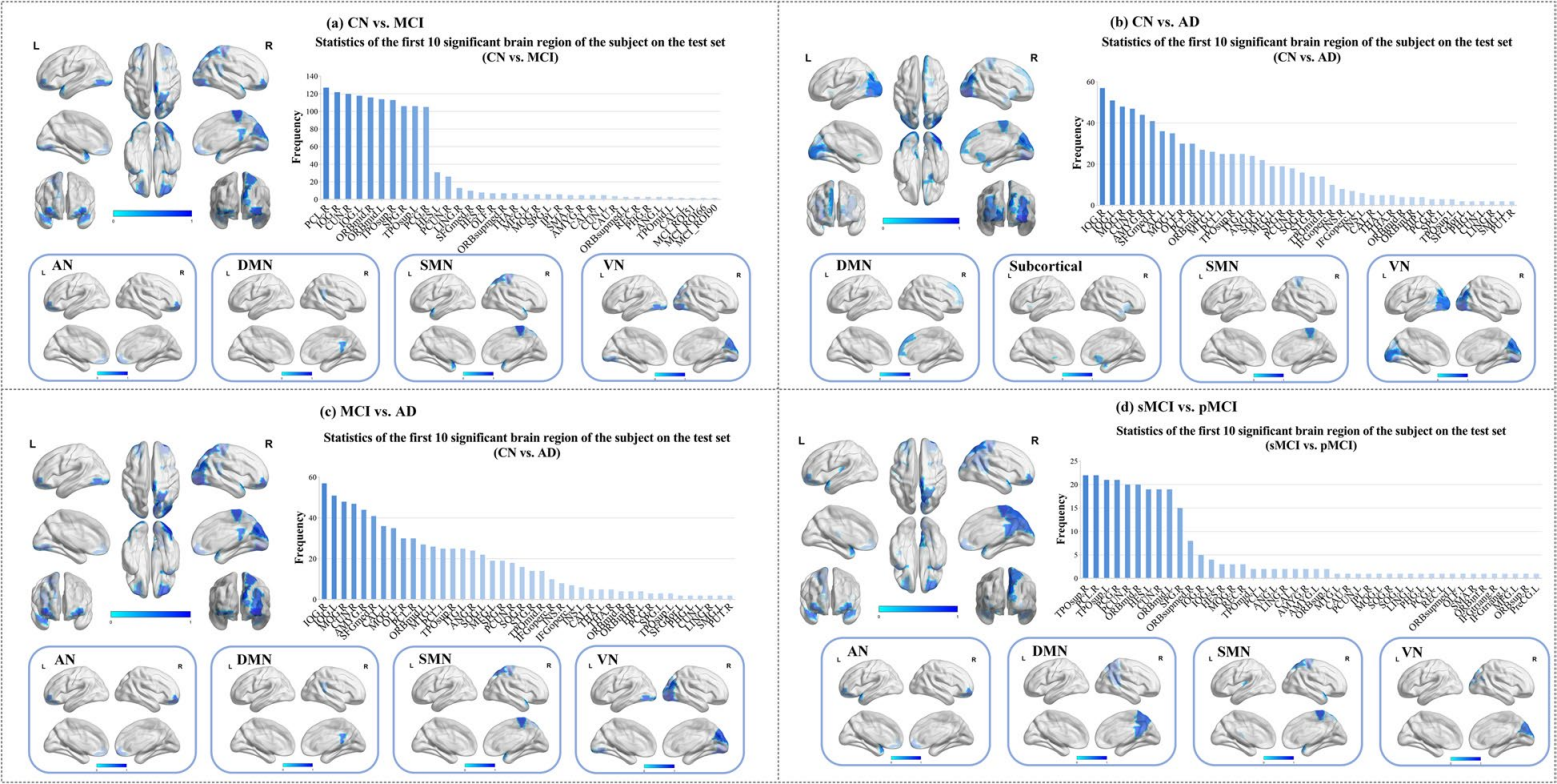
Visualization of ten important brain regions with the most damage. In the subfigure, the upper main image shows the location of important brain regions in multiple perspectives. The lower sub-image illustrates the distribution of these regions within the RSN

On the testing dataset, we identified the top ten brain regions with the highest frequency of severe damage (Table S6). These regions, namely the paracentral lobule, right inferior occipital gyrus, cuneus, left inferior occipital gyrus, right middle frontal gyrus, left middle frontal gyrus, right superior temporal gyrus, superior parietal gyrus, left superior temporal gyrus, and posterior cingulate gyrus, had the greatest impact on differentiating between MCI patients and the CN group. These regions primarily belong to the SMN, VN, AN, and DMN networks. AD patients exhibited abnormalities mainly in the VN, SUBN, DMN, and SMN networks compared to the CN group. Furthermore, the distinctive brain regions between AD and MCI were primarily distributed in the VN, SMN, DMN, and AN networks, while the different brain regions between sMCI and pMCI were primarily distributed in the SMN, AN, DMN, and VN networks, respectively. The visualization of these important brain regions is presented in Fig. 2.

### The features of the important brain regions predict the clinical progression of MCI to AD

**Fig. 3:**
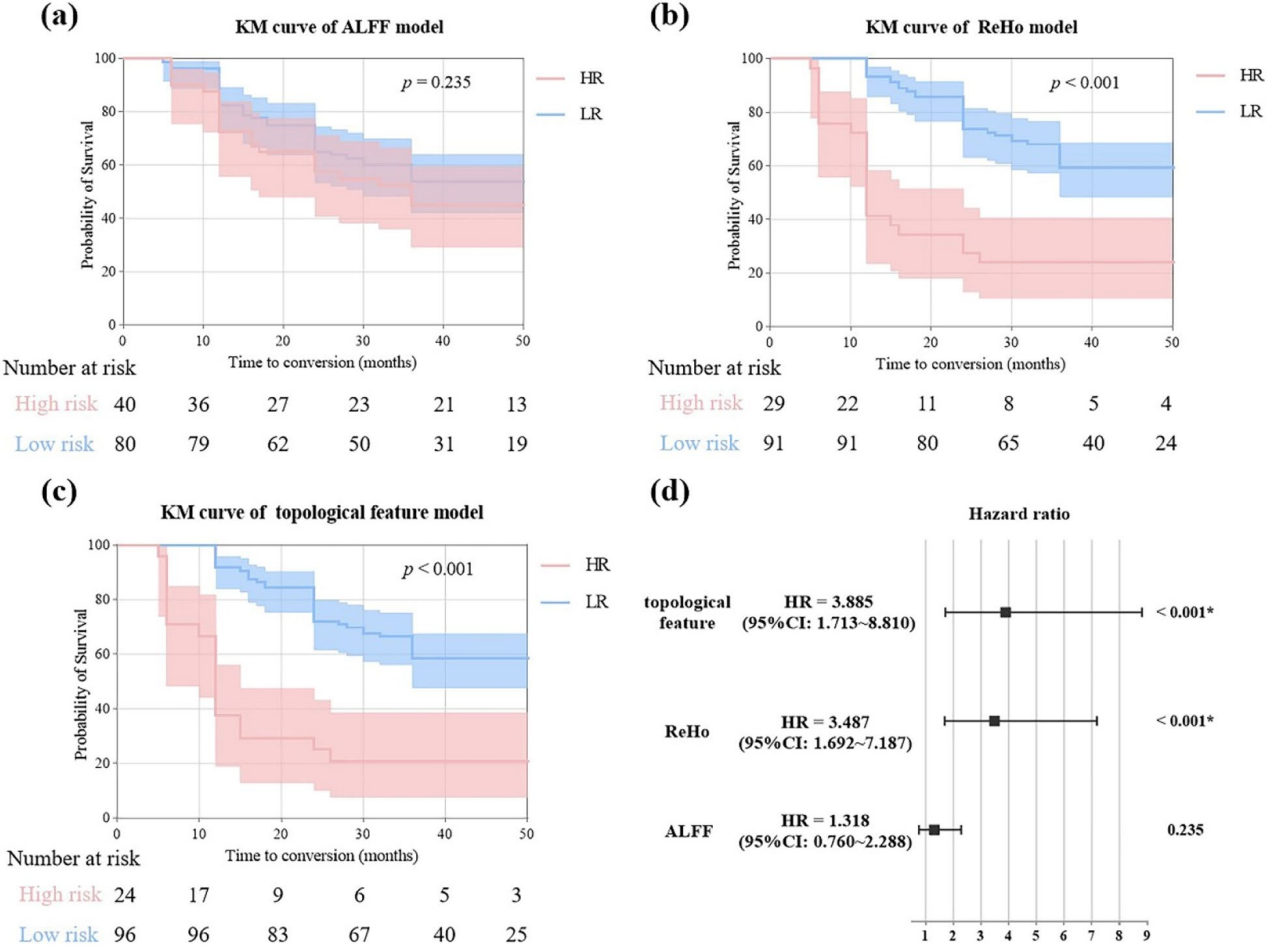
KM curves of (**a**) ALFF, (**b**) ReHo, and (**c**) the topological feature model. Forest plots (right of Fig. 3(d)) show HR and 95% CI for different predictors

The topological feature-based model demonstrated the strongest predictive capability (hazard ratio (HR) [95% confidence interval (CI)] = 3.885 [1.713–8.813], *p* < 0.001), followed by the ReHo-based model (HR [95% CI] = 3.487 [1.692–7.187], *p* < 0.001). In contrast, the ALFF-based model showed poor performance (HR [95% CI] = 1.318 [0.760–2.288], *p* = 0.235). As can be seen from Fig. 3, the ALFF model does not clearly differentiate between high-risk and low-risk individuals. The KM curves effectively differentiated individuals with high-risk and low-risk profiles in the ReHo, and topological feature models, especially the topological feature model. In the topological feature model, the risk ratio of the included predictors is shown in Figure S6.

### Correlation between topological characteristics and clinical measures

**Fig. 4:**
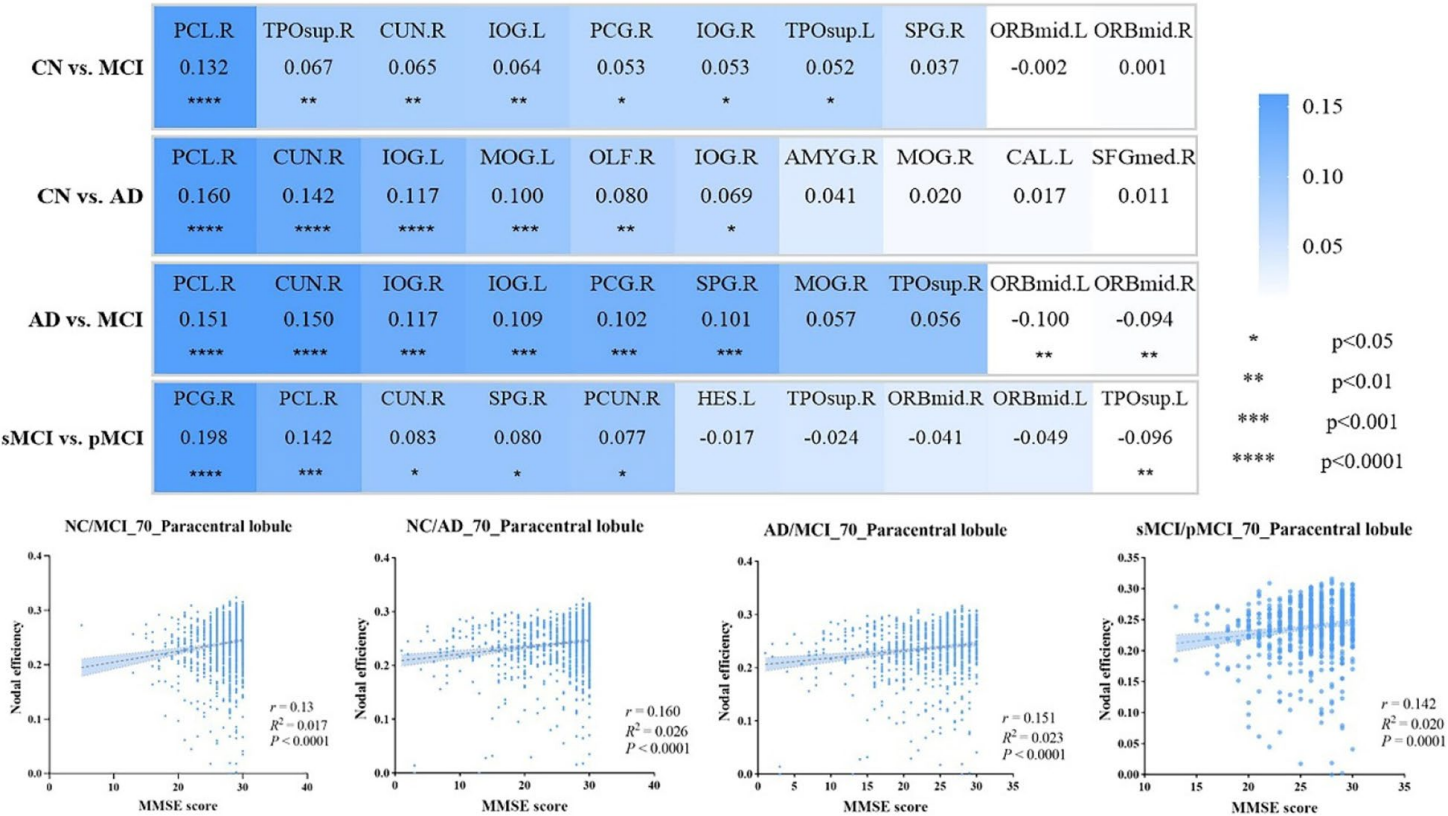
Correlation analysis between topological features (NE) and clinical indicators (MMSE score) in the important brain regions

In the important brain regions identified by STGC-GCAM, there is a significant correlation between the NE and MMSE score including paracentral lobule (r = 0.132, *p* < 0.0001), right inferior occipital gyrus (r = 0.053, *p* < 0.05), cuneus (r = 0.065, *p* < 0.01), left inferior occipital gyrus (r = 0.064, *p* < 0.01), right superior temporal gyrus (r = 0.067, *p* < 0.01), left superior temporal gyrus (r = 0.052, *p* < 0.05), and posterior cingulate gyrus (r = 0.053, *p* < 0.05) in both MCI and CN subjects. The NE in the right inferior occipital gyrus (r = 0.069, *p* < 0.05), left inferior occipital gyrus (r = 0.117, *p* < 0.0001), cuneus (r = 0.142, *p* < 0.0001), left middle occipital gyrus (r = 0.100, *p* < 0.001), right olfactory cortex (r = 0.080, *p* < 0.01), and paracentral lobule (r = 0.160, *p* < 0.0001) were significantly correlated with MMSE score in both AD and CN subjects. AD and MCI subjects showed significant correlations between NE in the cuneus (r = 0.150, *p* < 0.0001), right inferior occipital gyrus (r = 0.117, *p* < 0.001), paracentral lobule (r = 0.151, *p* < 0.0001), posterior cingulate gyrus (r = 0.102, *p* < 0.001), superior parietal gyrus (r = 0.101, *p* < 0.001), left inferior occipital gyrus (r = 0.109, *p* < 0.001), right middle frontal gyrus (r = -0.094, *p* < 0.01), and left middle frontal gyrus (r = -0.100, *p* < 0.01) and MMSE score. sMCI and pMCI subjects showed significant correlations between NE in the paracentral lobule (r = 0.142, *p* < 0.001), left superior temporal gyrus (r = -0.096, *p* < 0.01), posterior cingulate gyrus (r = 0.198, *p* < 0.0001), right precuneus (r = 0.077, *p* < 0.05), cuneus (r = 0.083, *p* < 0.05), and superior parietal gyrus (r = 0.080, *p* < 0.05) and MMSE scores (Fig. 4).

### The topological features of important brain regions mediated the effects of biomarkers on cognition

**Fig. 5:**
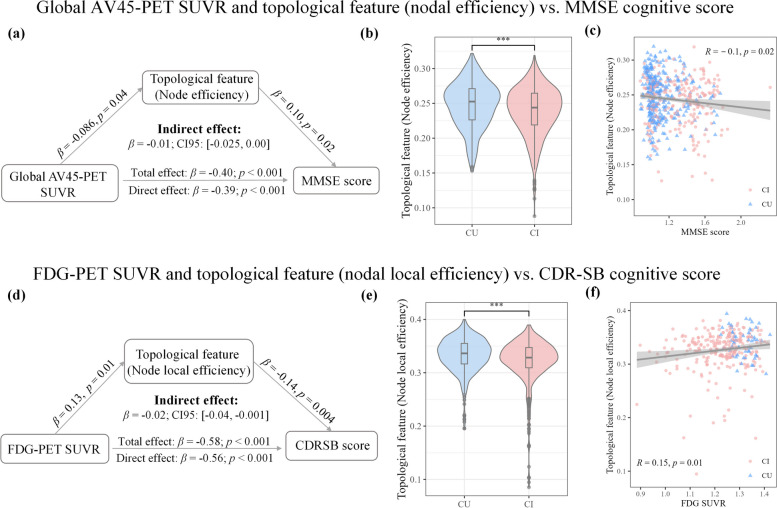
The topological characteristics of disease-related brain regions have a mediating effect on cognitive function. Please refer to the Jagust LABS PDF on LONI for calculations of global AV45-PET SUVR and FDG-PET SUVR

By selecting the topological features of important brain regions as predictor variables, we found a significant correlation between global AV45-PET SUVR and MMSE scores (β = -0.40, *p* < 0.001). This association was mediated by NE (bootstrapped average causal mediation effect: β = -0.01 [-0.025, 0.00], *p* < 0.001) (Fig. 5(a)), indicating a partial mediation effect. We observed significant differences in NE between the cognitively unimpaired (CU) and cognitively impaired (CI) groups (*p* < 0.001) (Fig. 5(b)). Furthermore, NE was significantly correlated with MMSE score (R = -0.10, *p* = 0.02) (Fig. 5(c)). Similarly, we found that NLE partially mediated the effects of average FDG-PET SUVR of angular, temporal, and posterior cingulate on participants’ cognition (bootstrapped average causal mediation effect: β = -0.02 [-0.04, -0.001], *p* < 0.001). There was a significant correlation between average FDG-PET SUVR of angular, temporal, and posterior cingulate and CDR-SB scores (β = -0.58, *p* < 0.001) (Fig. 5(d)). There were significant differences in NLE between the CU and CI groups (*p* < 0.001) (Fig. 5(e)). Additionally, NLE was significantly correlated with CDR-SB score (R = -0.15, *p* = 0.01) (Fig. 5(f)). No mediation effects were found between CSF TAU/PTAU, topological features, and neurocognitive scales. It is worth noting that significant correlations were observed between the topological features of important brain regions and CSF TAU (R = -0.21, *p* = 0.01) and CSF PTAU (R = -0.22, *p* < 0.01) (Figure S7).

## Discussion

This study presents the STGC-GCAM model for identifying novel FC biomarkers linked to AD. There are two innovative aspects of the model: (1) It utilizes ST-GCN to capture both temporal and spatial topological information within the data channels; (2) It adds a Grad-CAM module to extract critical node information on the graph, effectively characterizing the brain regions impacted by the disease. In comparison to prior research (Table S7), our model demonstrates superior performance across multi-center datasets. The effectiveness of the proposed model was ensured and evaluated through experiments on a large dataset from six different sites, focusing on AD and its subtypes. The identified important brain regions for AD, MCI, sMCI, and pMCI were primarily distributed in the DMN, SMN, and VN. Post hoc analyses demonstrated the excellent performance of the biomarkers in predictive experiments, supported by correlation and causal mediation effect analyses. These findings highlight the advantages of the STGC-GCAM model in personalized case identification and biomarker extraction, indicating its potential applicability in clinical settings.

While previous studies have demonstrated the disease identification capability and identification of important brain regions using GCN, our work presents significant advancements [13]. In this study, our model achieved an effective classification result on a large multi-center dataset by proposing the STGC-GCAM, which simultaneously considers the spatio-temporal features of fMRI data and the importance of nodes. Specifically, the ST-GCN model effectively handles spatiotemporal information by capturing temporal relationships, while Grad-CAM precisely localizes crucial nodes in the network using gradient information. Consequently, the STGC-GCAM approach enables the acquisition of comprehensive spatiotemporal feature representations and explanations of important nodes, facilitating a more thorough understanding of the decision-making process [43]. Notably, the integration of individual spatiotemporal features by ST-GCN ensures comprehensive consideration of individual heterogeneity. In contrast, conventional classifiers often process brain data by capturing functional connectivity values and converting them into vectors for analysis. This approach focuses on independent connections, overlooking the intricate relationships present within neighborhoods and the complex network structures of the brain. In summary, our STGC-GCAM model provides a unique and robust method for analyzing fMRI data, leveraging the strengths of both ST-GCN and Grad-CAM, thus enabling a nuanced examination of inter-individual variability and individual characteristics within the framework.

We trained the model on a multi-site dataset comprising 2272 participants, performing subgroup analysis across different stages of AD. Furthermore, we visualized the distribution of disease-specific features in the network using t-SNE (Figure S5). The important brain regions contributing significantly to classification were primarily found in the DMN, SEN, and VN. Previous fMRI studies have consistently reported widespread changes in DMN connectivity in individuals with MCI and AD [52–54]. Comparing our results to the control group, AD patients exhibited abnormal brain regions, including the right cuneus, right superior frontal gyrus, bilateral middle occipital gyrus, right amygdala, left calcarine fissure and surrounding cortex, left inferior occipital gyrus, right olfactory cortex, and right paracentral lobule. These findings are in line with previous research [2, 55–58]. Similarly, in the MCI groups, we observed FC abnormalities in the right paracentral lobule, bilateral orbital part of the middle frontal gyrus, bilateral superior temporal gyrus, right superior parietal gyrus, right cuneus, and right posterior cingulate gyrus, consistent with existing literature on FC abnormalities [14, 59, 60]. Additionally, we identified the inferior occipital gyrus as a region implicated in both AD and MCI, despite not being traditionally considered as a significant brain region associated with AD. Notably, Yang et al. [61] discovered abnormal connectivity in the inferior occipital gyrus among individuals with MCI and comorbid depression. This finding emphasizes the necessity for further research and individualized assessment of pathological features in specific populations.

To validate the pathophysiological significance of the identified brain regions, we performed correlation analyses between their topological characteristics and clinical features. Interestingly, after controlling for age, sex, education level, and APOE ε4 status, we observed that the topological features of these regions mediated the relationship between neurodegenerative biomarkers (AV45-PET and FDG-PET SUVR) and neurocognitive scale scores. For the diagnosis of AD, the most validated neuroimaging biomarkers include medial temporal lobe atrophy on MRI, posterior cingulate and temporoparietal hypometabolism on FDG-PET, and cortical Aβ deposition on amyloid-PET imaging [62]. AV45-PET is particularly useful for ruling out AD, while FDG-PET aids in the differential diagnosis of neurodegenerative diseases, prediction of short-term clinical outcomes, and staging of neurodegenerative processes. In early AD, a fMRI study demonstrated that the extent of FC abnormalities is associated with the degree of Aβ pathology (measured by CSF and PET), but not tau pathology (measured by CSF) [63]. This suggests that Aβ pathology, rather than tau pathology, primarily drives functional connectome changes in the early stages of AD. Our findings revealed a close relationship between FC, FDG-PET, AV45-PET, and cognitive impairment in individuals with AD.

However, our study has some limitations. Despite the utilization of data from multiple imaging centers, limitations in sample information exist, which restricts the generalizability of our findings. To overcome this limitation, future enhancements in data collection are required, particularly in Cohort III where follow-up data was incomplete. In addition, while we examined FC abnormalities, our analysis focused solely on affected brain regions rather than connectivity patterns. Future research should investigate changes in FC throughout the progression of AD to identify more accurate biomarkers for AD. Then, our study exclusively considered resting-state functional networks. To gain a comprehensive understanding of AD pathophysiology, future studies could incorporate other types of networks using different imaging modalities, such as gray matter covariance networks, white matter connectivity networks, or combinations thereof. This would provide a more comprehensive perspective on AD identification. Future research should address these limitations by improving data collection, examining connectivity patterns, and incorporating different types of networks and imaging modalities to enhance the identification of AD from the perspective of AD pathophysiology. Moreover, we exclusively utilized fMRI data features as the model input in this study. Our future research plan is to integrate more clinical data into the STGC-GCAM model, such as age, sex, education, APOE4, CSF TAU/PTAU, etc., which may improve the classification accuracy of the model.

## Conclusion

This study introduces the STGC-GCAM framework, which enables the identification of a novel biomarker and facilitates early diagnosis of AD using a large multi-site fMRI dataset. The results underscore the potential of deep learning methods in providing objective and stable biomarkers that can aid in the early detection of AD.

### Supplementary Information

**Additional file 1. **Supplementary data related to this article can be found.
