# Supplementary material for: A novel spatiotemporal graph convolutional network framework for functional connectivity biomarkers identification of Alzheimer’s disease
Source: Alzheimers Res Ther. 2024 Mar 14;16:60. doi: 10.1186/s13195-024-01425-8 (PMC10938710; doi:10.1186/s13195-024-01425-8)
Supplement: Supplementary file 1 — Additional file 1. Supplementary data related to this article can be found. [file 13195_2024_1425_MOESM1_ESM.docx]

## Gradient-weighted class activation mapping

Assuming that the feature map of the convolutional unit of the last layer of space-time graph is $\mathbf{Z}_{\mathrm{stg}}\boldsymbol{\in}\mathbb{R}^{\mathbf{C}\boldsymbol{\times}\mathbf{T}\boldsymbol{\times}\mathbf{N}}$, the classification probability obtained through the fully connected layer is $y^{\theta}$, and the calculation formula of the class activation map value of category theta in channel C is as follows:

$$\begin{aligned} \boldsymbol{\alpha}^{\theta, C}=\underset{\mathrm{GAP}}{\underbrace{\frac{1}{\mathrm{NT}}\sum_{n=1}^{N} \sum_{t=1}^{T}}}\overset{\mathrm{gradients}}{\overbrace{\frac{\delta y^{\theta}}{\delta\mathbf{Z}_{\mathrm{stg}}^{t,n}}}}\#\left( 1 \right) \end{aligned}$$

The formula for calculating the gradient-based feature activation heatmap on the last layer of convolution is:

$$\begin{aligned} \mathbf{H}_{\mathrm{ST}}^{\theta}=\mathrm{ReLu}\underset{linear combination}{\underbrace{\left( \sum_{C} \boldsymbol{\alpha}^{\theta, C}\mathbf{Z}_{\mathrm{stg}}^{C} \right)}}\#\left( 2 \right) \end{aligned}$$

Through STGC-GCAM, we can obtain category-specific heatmaps in specified network layers, from which we can understand the importance of different nodes for different categories. To create the final node-class importance heatmap, the node-map values for each class are obtained and averaged over all correctly classified data for individual nodes. Let M be the amount of data correctly classified by category $\theta$, then the definition formula of the spatio-temporal joint importance degree mapping of node n is:

$$\begin{aligned} \mathbf{L}_{\mathrm{ST}}^{\theta, n}=\frac{1}{M}\sum_{m=1}^{M} \mathbf{H}_{\mathrm{ST}}^{\theta, n}\#\left( 3 \right) \end{aligned}$$

## Training strategy

The model parameters to be tuned include adjacency matrix binarization threshold (THR), one-dimensional time convolution kernel size (KS), random dropout rate (DR), initial learning rate (LR), l2 regularization parameters (L2) and batch size (BS). The optimizer of the model uses Adam. In order to deal with the imbalance between positive and negative data samples, we use a weighted cross-entropy function whose formula is as follows:

$$\begin{aligned} L=\frac{1}{N}\sum_{i}^{N} \left[ \omega_{i}^{1}\times y_{\mathrm{true}}\times\text{log}\left( y_{\mathrm{pred}} \right)+\omega_{i}^{0}\times\left( 1-y_{\mathrm{true}} \right)\times\log\left( 1-y_{\mathrm{pred}} \right) \right]\boldsymbol{\#}\left( 4 \right) \end{aligned}$$

Provide a larger penalty coefficient for samples with fewer categories; provide a smaller penalty coefficient for samples with more categories. The training process of the model in this research lasts for 50 epochs. STGC-GCAM is implemented in Python using the PyTorch deep learning framework, and experiments are performed on an NVIDIA TITAN Xp graphics card.

**Table S1:** The algorithm flow of STGC-GCAM.


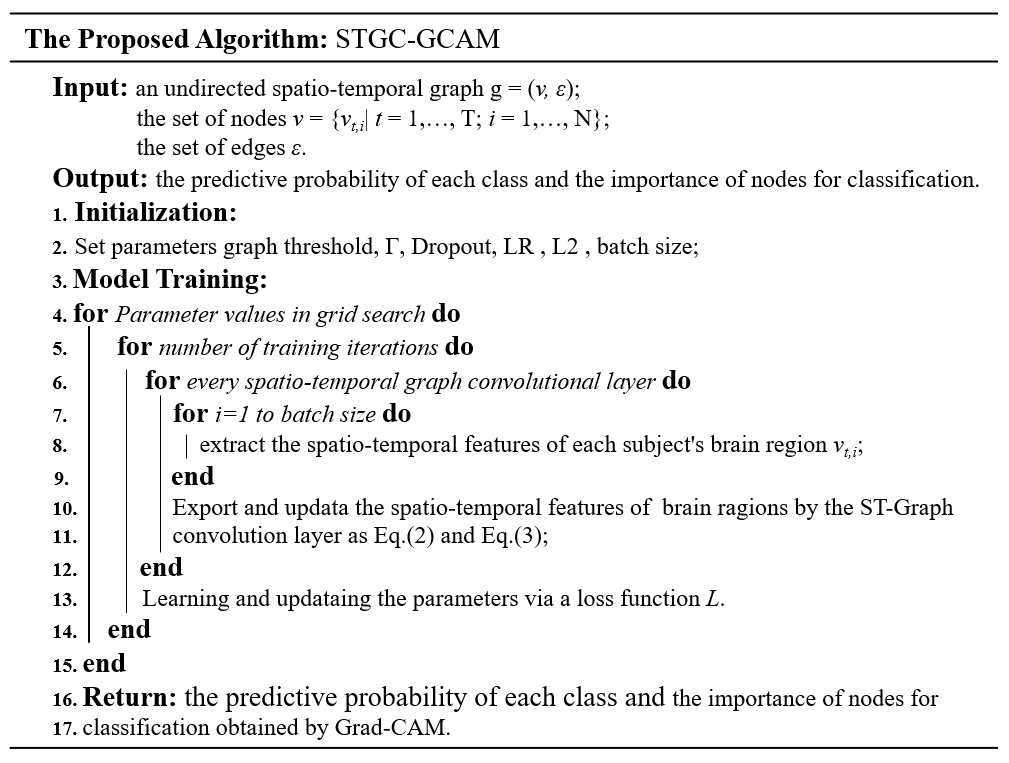


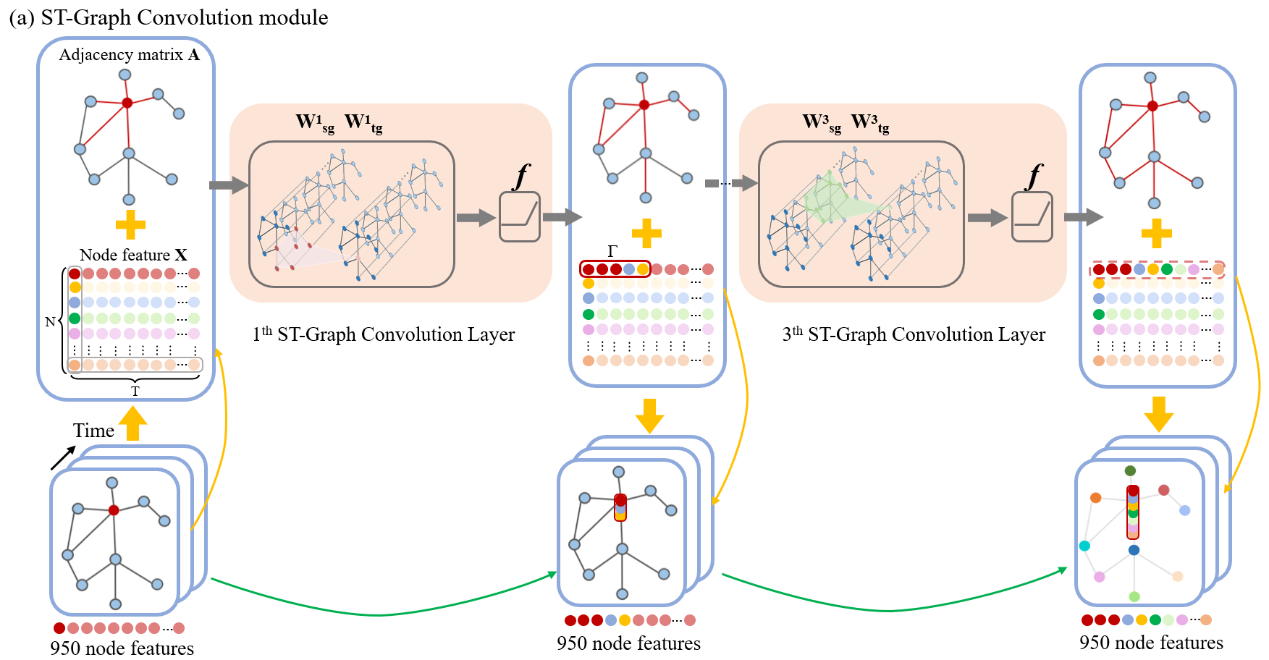


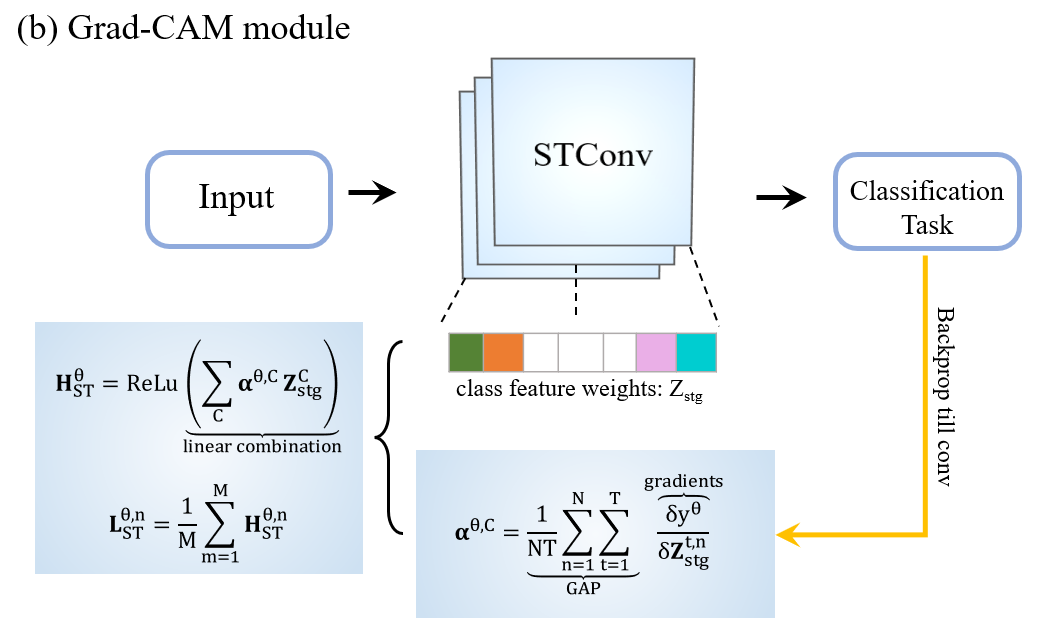


**Figure S1:** Detailed architecture of important components in STGC-GCAM, including ST-GCN and Grad-CAM module. (a) STGCN module algorithm flow chart, it includes space convolution and time convolution. (b) Flow chart of Grad-CAM module, it is based on ST-GCN module.

**Table S2:** Hyperparameter selection of STGC-GCAM on different data datasets.

| CN vs. MCI | | | | CN vs. AD | | | MCI vs. AD | | | | sMCI vs. pMCI | | |
| --- | --- | --- | --- | --- | --- | --- | --- | --- | --- | --- | --- | --- | --- |
| **Site** | **THR** | **LR** | **L2** | **THR** | **LR** | **L2** | **THR** | **LR** | **L2** | **THR** | | **LR** | **L2** |
| **Site1** | 60 | 0.001 | 0.001 | 55 | 0.001 | 0.001 | 80 | 0.01 | 0.001 | 75 | | 0.001 | 0.001 |
| **Site2** | 50 | 0.001 | 0.001 | 50 | 0.001 | 0.001 | 65 | 0.001 | 0.001 | 75 | | 0.001 | 0.001 |
| **Site3** | 70 | 0.001 | 0.01 | 55 | 0.001 | 0.001 | 70 | 0.001 | 0.1 | 85 | | 0.001 | 0.001 |
| **Site4** | 70 | 0.01 | 0.001 | 50 | 0.01 | 0.001 | 65 | 0.001 | 0.1 | 75 | | 0.001 | 0.1 |
| **Site5** | 55 | 0.001 | 0.01 | 55 | 0.01 | 0.001 | 70 | 0.001 | 0.01 | - | | - | - |
| **Site6** | 65 | 0.001 | 0.001 | 60 | 0.001 | 0.001 | 65 | 0.001 | 0.1 | - | | - | - |
| **ALL** | 70 | 0.001 | 0.001 | 80 | 0.01 | 0.001 | 80 | 0.001 | 0.001 | 85 | | 0.01 | 0.1 |

Note: On all datasets, KS = 7, BS = 30, DR = 0.2. ALL = site1+site2+site3+site4+site5+site6.

Abbreviations: BS, batch size; DR, random dropout rate; KS, time convolution kernel size; LR, initial learning rate; L2: l2 regularization parameters; THR, adjacency matrix binarization threshold.

## Participants

**Cohort I:**

The inclusion criteria of MCI patients enrolled from ADNI database were as follows: (1) all patients with sMCI and pMCI underwent fMRI scans and clinical diagnoses at the baseline visit and were followed up for at least 3 years; (2) sMCI patients who had not converted to AD at follow-up and pMCI patients who had converted to AD within the follow-up interval; and (3) participants with a bidirectional change in diagnosis (MCI to AD, and back to MCI) within the follow-up period were excluded.

**Cohort II:**

**Inclusion criteria**

The inclusion criteria are as follows: (1) 50–79 years old, right-handed and Mandarin-speaking subjects; (2) presence of self-perceived continuous cognitive decline compared with previous normal status and unrelated to an acute event; (3) concerns (worries) associated with memory complaint; and (4) failure to meet the criteria for MCI or dementia.

**The diagnostic criteria**

NC: The inclusion criteria are as follows: (1) 50–79 years old, right-handed and Mandarin-speaking subjects; (2) presence of self-perceived continuous cognitive decline compared with previous normal status and unrelated to an acute event; (3) concerns (worries) associated with memory complaint; and (4) failure to meet the criteria for MCI or dementia.

MCI: Participants were diagnosed with MCI if they met any one of the following three criteria and failed to meet the criteria for dementia: (1) having impaired scores (defined as >1.0 SD below the age/education-corrected normative means) on both measures in at least one cognitive domain (memory, language, or speed/executive function); (2) having impaired scores in each of the three cognitive domains (memory, language, or speed/ executive function); and (3) Functional Activities Questionnaire≥9. Individuals with memory complaints and objective memory decline were considered as aMCI patients [1], and those without significant deficits in the memory domain were regarded as naMCI patients.

AD: The diagnosis of AD dementia is based on the Diagnostic and Statistical Manual of Mental Disorders, fifth edition, and the diagnostic guidelines for dementia due to AD delivered by the National Institute on Ageing–Alzheimer’s Association workgroups[2], and a total CDR score ≥1.

**Cohort III:**

All of the subjects were diagnosed by experienced clinicians. The concept of MCI refers to a stage between normal aging and dementia, including but not limited to subjective perception of cognitive decline and abnormal neuropsychological testing results. A redefined AD criteria is proposed by the National Institute on Aging (NIA) committee.

**Exclusion criteria**

The exclusion criteria are as follows: (1) History of stroke. (2) Current major psychiatric diagnoses such as severe depression and anxiety. (3) Other neurological conditions that could cause cognitive decline (eg, brain tumours, Parkinson’s disease, encephalitis, or epilepsy) rather than AD spectrum disorders. (4) Other diseases that could cause cognitive decline (eg, thyroid dysfunction, severe anaemia, syphilis, or HIV). (5) History of psychosis or congenital mental developmental delay. (6) Cognitive decline caused by traumatic brain injury. (7) Inability to complete the study protocol or presence of contraindications for MRI.

**Figure S2:** Flowchart of the inclusion and exclusion of ADNI cohort participants.

**Figure S3:** Flowchart of the inclusion and exclusion of Xuanwu cohort participants.

**Figure S4:** Flowchart of the inclusion and exclusion of Tongji cohort participants.

## Neuroimaging acquisition and preprocessing

**Table S3:** Scanner equipment and acquisition parameters for each site.

| **Cohort** | **Site** | **Scanner** | **Field of view (mm^2^)** | **TR (ms)** | **TE (ms)** | **Flip angle (°)** | **Thickness (mm)** | **Gap (mm)** | **Number of slices** | **Number of Volumes** | **Voxel size (mm^3^)** |
| --- | --- | --- | --- | --- | --- | --- | --- | --- | --- | --- | --- |
| ADNI | 1 | Philips Achieva (3T) | - | 3000 | 30 | 80 | 3.3 | - | 48 | 140 | 3.3×3.3×3.3 |
|  | 2 | Siemens Prisma_fit (3T) | - | 3000 | 30 | 90 | 3.4 | - | 48 | 197 | 3.4×3.4×3.4 |
| Xuanwu | 3 | Siemens TrioTim (3T) | 240×240 | 2000 | 40 | 90 | 4.0 | 1.0 | 28 | 239 | 4×4×5 |
|  | 4 | GE Sigma PET/MR (3T) | 224×224 | 2000 | 30 | 90 | 4.0 | 1.0 | 28 | 240 | 3.5×3.5×4.0 |
| Tongji | 5 | Siemens Verio  (3T) | 224×224 | 2000 | 30 | 90 | 3.6 | - | 31 | 240 | 3.5×3.5×3.6 |
|  | 6 | Siemens Prisma_fit (3T) | 224×224 | 500 | 30 | 60 | 3.5 | - | 35 | 960 | 3.5×3.5×3.5 |

Abbreviations: TR, repetition time; TE, echo time.

## Clinical application of STGC-GCAM

**Table S4:** Numbering of brain regions in the Automated Anatomical Labeling (AAL) template and their assignment to resting-state networks.

| Number | Name | Acronyms | RSN |
| --- | --- | --- | --- |
| 1 | Precental gyrus | PreCG.L | Sensorimoor |
| 2 | Precental gyrus | PreCG.R | Sensorimoor |
| 3 | Superior frontal gyrus, dorsolateral | SFGdor.L | Default mode |
| 4 | Superior frontal gyrus, dorsolateral | SFGdor.R | Default mode |
| 5 | Superior frontal gyrus, orbital part | ORBsup.L | Attention |
| 6 | Superior frontal gyrus, orbital part | ORBsup.R | Default mode |
| 7 | Middle frontal gyrus | MFG.L | Attention |
| 8 | Middle frontal gyrus | MFG.R | Attention |
| 9 | Middle frontal gyrus, orbital part | ORBmid.L | Attention |
| 10 | Middle frontal gyrus, orbital part | ORBmid.R | Attention |
| 11 | Inferior frontal gyrus, opercular part | IFGoperc.L | Attention |
| 12 | Inferior frontal gyrus, opercular part | IFGoperc.R | Attention |
| 13 | Inferior frontal gyrus, triangular part | IFGtriang.L | Attention |
| 14 | Inferior frontal gyrus, triangular part | IFGtriang.R | Attention |
| 15 | Inferior frontal gyrus, orbital part | ORBinf.L | Attention |
| 16 | Inferior frontal gyrus, orbital part | ORBinf.R | Attention |
| 17 | Rolandic operculum | ROL.L | Sensorimoor |
| 18 | Rolandic operculum | ROL.R | Sensorimoor |
| 19 | Supplementary motor area | SMA.L | Attention |
| 20 | Supplementary motor area | SMA.R | Attention |
| 21 | Olfactory cortex | OLF.L | Subcortical |
| 22 | Olfactory cortex | OLF.R | Subcortical |
| 23 | Superior frontal gyrus, medial | SFGmed.L | Default mode |
| 24 | Superior frontal gyrus, medial | SFGmed.R | Default mode |
| 25 | Superior frontal gyrus, medial orbital | ORBsupmed.L | Default mode |
| 26 | Superior frontal gyrus, medial orbital | ORBsupmed.R | Default mode |
| 27 | Gyrus rectus | REC.L | Default mode |
| 28 | Gyrus rectus | REC.R | Default mode |
| 29 | Insula | INS.L | Sensorimoor |
| 30 | Insula | INS.R | Sensorimoor |
| 31 | Anterior cingulate and paracingulate gyri | ACG.L | Default mode |
| 32 | Anterior cingulate and paracingulate gyri | ACG.R | Default mode |
| 33 | Median cingulate and paracingulate gyri | DCG.L | Subcortical |
| 34 | Median cingulate and paracingulate gyri | DCG.R | Subcortical |
| 35 | Posterior cingulate gyrus | PCG.L | Default mode |
| 36 | Posterior cingulate gyrus | PCG.R | Default mode |
| 37 | Hippocampus | HIP.L | Subcortical |
| 38 | Hippocampus | HIP.R | Subcortical |
| 39 | Parahippocampal gyrus | PHG.L | Subcortical |
| 40 | Parahippocampal gyrus | PHG.R | Subcortical |
| 41 | Amygdala | AMYG.L | Subcortical |
| 42 | Amygdala | AMYG.R | Subcortical |
| 43 | Calcarine fissure and surrounding cortex | CAL.L | Visual |
| 44 | Calcarine fissure and surrounding cortex | CAL.R | Visual |
| 45 | Cuneus | CUN.L | Visual |
| 46 | Cuneus | CUN.R | Visual |
| 47 | Lingual gyrus | LING.L | Visual |
| 48 | Lingual gyrus | LING.R | Visual |
| 49 | Superior occipital gyrus | SOG.L | Visual |
| 50 | Superior occipital gyrus | SOG.R | Visual |
| 51 | Middle occipital gyrus | MOG.L | Visual |
| 52 | Middle occipital gyrus | MOG.R | Visual |
| 53 | Inferior occipital gyrus | IOG.L | Visual |
| 54 | Inferior occipital gyrus | IOG.R | Visual |
| 55 | Fusiform gyrus | FFG.L | Visual |
| 56 | Fusiform gyrus | FFG.R | Visual |
| 57 | Postcentral gyrus | PoCG.L | Sensorimoor |
| 58 | Postcentral gyrus | PoCG.R | Sensorimoor |
| 59 | Superior parietal gyrus | SPG.L | Sensorimoor |
| 60 | Superior parietal gyrus | SPG.R | Sensorimoor |
| 61 | Inferior parietal, but supramarginal and angular gyri | IPL.L | Attention |
| 62 | Inferior parietal, but supramarginal and angular gyri | IPL.R | Attention |
| 63 | Supramarginal gyrus | SMG.L | Sensorimoor |
| 64 | Supramarginal gyrus | SMG.R | Sensorimoor |
| 65 | Angular gyrus | ANG.L | Attention |
| 66 | Angular gyrus | ANG.R | Attention |
| 67 | Precuneus | PCUN.L | Default mode |
| 68 | Precuneus | PCUN.R | Default mode |
| 69 | Paracentral lobule | PCL.L | Sensorimoor |
| 70 | Paracentral lobule | PCL.R | Sensorimoor |
| 71 | Caudate nucleus | CAU.L | Subcortical |
| 72 | Caudate nucleus | CAU.R | Subcortical |
| 73 | Lenticular nucleus, putamen | PUT.L | Subcortical |
| 74 | Lenticular nucleus, putamen | PUT.R | Subcortical |
| 75 | Lenticular nucleus, pallidum | PAL.L | Subcortical |
| 76 | Lenticular nucleus, pallidum | PAL.R | Subcortical |
| 77 | Thalamus | THA.L | Subcortical |
| 78 | Thalamus | THA.R | Subcortical |
| 79 | Heschl gyrus | HES.L | Sensorimoor |
| 80 | Heschl gyrus | HES.R | Sensorimoor |
| 81 | Superior temporal gyrus | STG.L | Sensorimoor |
| 82 | Superior temporal gyrus | STG.R | Sensorimoor |
| 83 | Temporal pole: superior temporal gyrus | TPOsup.L | Attention |
| 84 | Temporal pole: superior temporal gyrus | TPOsup.R | Sensorimoor |
| 85 | Middle temporal gyrus | MTG.L | Default mode |
| 86 | Middle temporal gyrus | MTG.R | Default mode |
| 87 | Temporal pole: middle temporal gyrus | TPOmid.L | Subcortical |
| 88 | Temporal pole: middle temporal gyrus | TPOmid.R | Subcortical |
| 89 | Inferior temporal gyrus | ITG.L | Attention |
| 90 | Inferior temporal gyrus | ITG.R | Default mode |
| 91 |  | Cerebelum_Crus1_L |  |
| 92 |  | Cerebelum_Crus1_R |  |
| 93 |  | Cerebelum_Crus2_L |  |
| 94 |  | Cerebelum_Crus2_R |  |
| 95 |  | Cerebelum_3_L |  |
| 96 |  | Cerebelum_3_R |  |
| 97 |  | Cerebelum_4_5_L |  |
| 98 |  | Cerebelum_4_5_R |  |
| 99 |  | Cerebelum_6_L |  |
| 100 |  | Cerebelum_6_R |  |
| 101 |  | Cerebelum_7b_L |  |
| 102 |  | Cerebelum_7b_R |  |
| 103 |  | Cerebelum_8_L |  |
| 104 |  | Cerebelum_8_R |  |
| 105 |  | Cerebelum_9_L |  |
| 106 |  | Cerebelum_9_R |  |
| 107 |  | Cerebelum_10_L |  |
| 108 |  | Cerebelum_10_R |  |
| 109 |  | Vermis_1_2 |  |
| 110 |  | Vermis_3 |  |
| 111 |  | Vermis_4_5 |  |
| 112 |  | Vermis_6 |  |
| 113 |  | Vermis_7 |  |
| 114 |  | Vermis_8 |  |
| 115 |  | Vermis_9 |  |
| 116 |  | Vermis_10 |  |

## Result

### Evaluation of the performance of STGC-GCAM models


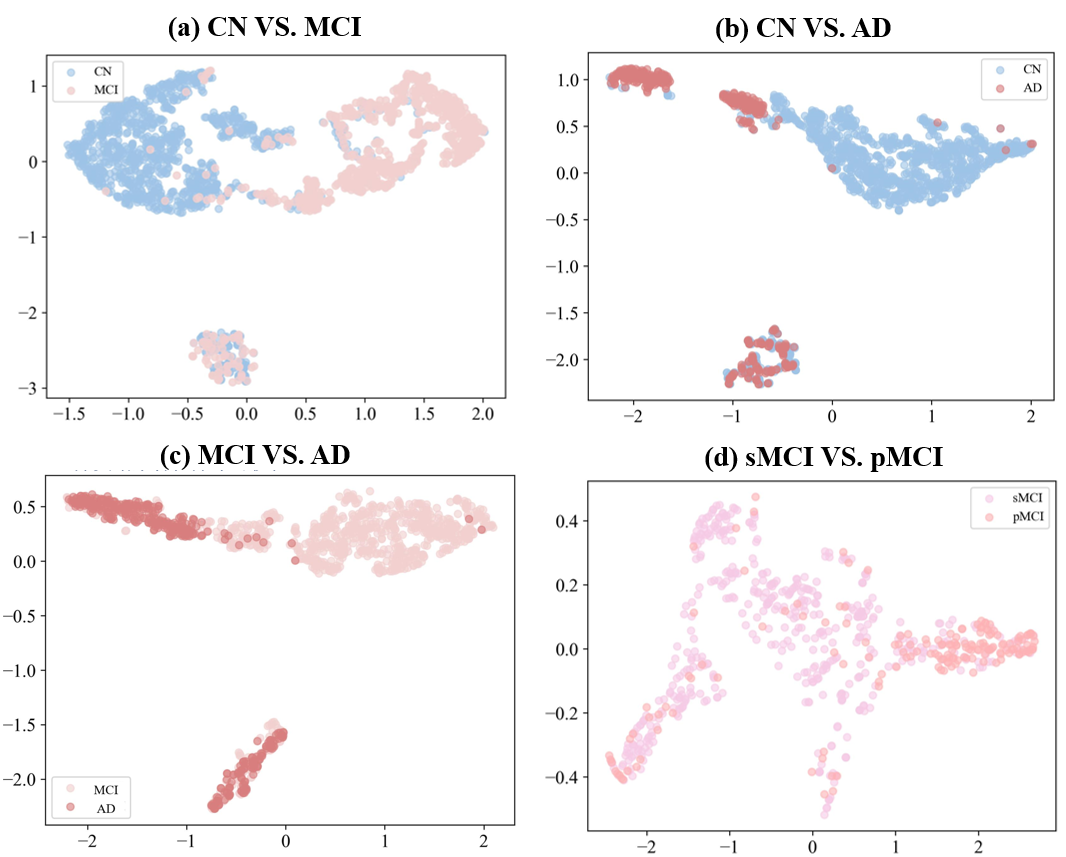


**Figure S5:** Visualization of the feature distribution using t-SNE, embedded in a space of dimension 2D. Each scatter plot represents the visualization of the network features for both categories, which are (a) CN vs. MCI, (b) CN vs. AD, (c) MCI vs. AD, and (d) sMCI vs. pMCI.

**Table S5:** Single-site classification performance of the STGC-GCAM model across 6 sites for different classification tasks.

| CN vs. MCI | | | | | CN vs. AD | | | | MCI vs. AD | | | | sMCI vs. pMCI | | | |
| --- | --- | --- | --- | --- | --- | --- | --- | --- | --- | --- | --- | --- | --- | --- | --- | --- |
| **Site** | **ACC** | **SEN** | **SPE** | **AUC** | **ACC** | **SEN** | **SPE** | **AUC** | **ACC** | **SEN** | **SPE** | **AUC** | **ACC** | **SEN** | **SPE** | **AUC** |
| **Site1** | 0.84±0.01 | 0.77±0.01 | 0.94±0.01 | 0.89±0.01 | 0.66±0.01 | 0.52±0.02 | 0.79±0.03 | 0.71±0.01 | 0.80±0.01 | 0.90±0.01 | 0.75±0.01 | 0.87±0.01 | 0.70±0.01 | 0.68±0.01 | 0.71±0.01 | 0.68±0.01 |
| **Site2** | 0.72±0.004 | 0.61± 0.01 | 0.77±0.01 | 0.75±0.003 | 0.80±0.01 | 0.80±0.02 | 0.80±0.01 | 0.86±0.01 | 0.68±0.01 | 0.54±0.04 | 0.72±0.03 | 0.61±0.02 | 0.81±0.01 | 0.62±0.02 | 0.83±0.01 | 0.72±0.01 |
| **Site3** | 0.62± 0.01 | 0.53±0.03 | 0.70±0.02 | 0.62±0.01 | 0.79±0.02 | 0.58±0.02 | 0.85±0.02 | 0.70±0.01 | 0.70±0.02 | 0.50±0.06 | 0.76±0.05 | 0.62±0.01 | 0.66±0.01 | 0.68±0.04 | 0.66±0.03 | 0.67±0.02 |
| **Site4** | 0.81± 0.01 | 0.44±0.03 | 0.88±0.01 | 0.73±0.01 | 0.93±0.01 | 0.73±0.03 | 0.94±0.02 | 0.91±0.01 | 0.68±0.03 | 0.55±0.08 | 0.73±0.07 | 0.64±0.04 | 0.94±0.01 | 1.00±0.00 | 0.91±0.01 | 0.98±0.01 |
| **Site5** | 0.66±0.01 | 0.65±0.03 | 0.67±0.04 | 0.61±0.02 | 0.68±0.02 | 0.88±0.02 | 0.52±0.04 | 0.77±0.01 | 0.63±0.01 | 0.66±0.04 | 0.60±0.05 | 0.56±0.02 | - | - | - | - |
| **Site6** | 0.68±0.01 | 0.52±0.04 | 0.83± 0.02 | 0.65±0.01 | 0.73±0.01 | 0.77±0.04 | 0.71±0.02 | 0.74±0.02 | 0.61±0.02 | 0.79±0.05 | 0.49±0.06 | 0.60±0.01 | - | - | - | - |

Values are reported in terms of mean±SD. Abbreviations: ACC, accuracy; SEN, specificity; SPE, specificity; AUC, area under receiver operating characteristic curve.

### Imaging marker identification and validation

**Table S6:** The top ten pathological brain regions with the most damage.

| **Rank** | **MCI vs. CN** | | | |  | **AD vs. CN** | | | |  | | **AD vs.MCI** | | | |  | | **sMCI vs. pMCI** | | | | |
| --- | --- | --- | --- | --- | --- | --- | --- | --- | --- | --- | --- | --- | --- | --- | --- | --- | --- | --- | --- | --- | --- | --- |
|  | Brain Region | Frequency | Weight (mean ±std) | RSN | Brain Region | | Frequency | Weight (mean ±std) | RSN | | Brain Region | | Frequency | Weight (mean ±std) | RSN | | Brain Region | | Frequency | Weight (mean ±std) | RSN |  |
| 1 | 70_ PCL.R | 127 | 0.92±0.2 | SMN | 54_ IOG.R | | 57 | 0.95±0.1 | VN | | 46_ CUN.R | | 76 | 0.85±0.03 | VN | | 84_ TPOsup.R | | 22 | 0.74±0.2 | SMN |  |
| 2 | 54_ IOG.R | 122 | 0.82±0.2 | VN | 53_ IOG.L | | 51 | 0.74±0.1 | VN | | 54_ IOG.R | | 76 | 0.98±0.04 | VN | | 70_ PCL.R | | 22 | 0.85±0.3 | SMN |  |
| 3 | 46_ CUN.R | 120 | 0.71±0.1 | VN | 52_ MOG.R | | 48 | 0.70±0.1 | VN | | 70_ PCL.R | | 76 | 0.85±0.1 | SMN | | 83_ TPOsup.L | | 21 | 0.67±0.2 | AN |  |
| 4 | 53_ IOG.L | 118 | 0.78±0.2 | VN | 46_ CUN.R | | 47 | 0.66±0.2 | VN | | 36_ PCG.R | | 74 | 0.76±0.1 | DMN | | 36_ PCG.R | | 21 | 0.75±0.2 | DMN |  |
| 5 | 10_ ORBmid.R | 116 | 0.73±0.2 | AN | 42_ AMYG.R | | 44 | 0.68±0.2 | SUBN | | 60_ SPG.R | | 74 | 0.78±0.1 | SMN | | 68_ PCUN.R | | 20 | 0.71±0.2 | DMN |  |
| 6 | 09_ ORBmid.L | 114 | 0.75±0.2 | AN | 24_ SFGmed.R | | 41 | 0.47±0.3 | DMN | | 53_ IOG.L | | 52 | 0.79±0.04 | VN | | 10_ ORBmid.R | | 20 | 0.71±0.3 | AN |  |
| 7 | 84_ TPOsup.R | 113 | 0.72±0.1 | SMN | 43_ CAL.L | | 36 | 0.60±0.2 | VN | | 10_ ORBmid.R | | 51 | 0.82±0.05 | AN | | 79_ HES.L | | 19 | 0.66±0.2 | SMN |  |
| 8 | 60_ SPG.R | 106 | 0.67±0.1 | SMN | 51_ MOG.L | | 35 | 0.61±0.1 | VN | | 09_ ORBmid.L | | 50 | 0.73±0.03 | AN | | 46_ CUN.R | | 19 | 0.68±0.2 | VN |  |
| 9 | 83_ TPOsup.L | 106 | 0.69±0.1 | SMN | 22_ OLF.R | | 30 | 0.56±0.2 | SUBN | | 52_ MOG.R | | 49 | 0.70±0.02 | VN | | 09_ ORBmid.L | | 19 | 0.64±0.3 | AN |  |
| 10 | 36_ PCG.R | 105 | 0.69±0.1 | DMN | 70_ PCL.R | | 30 | 0.62±0.3 | SMN | | 84_ TPOsup.R | | 46 | 0.74±0.04 | SMN | | 60_ SPG.R | | 15 | 0.61±0.2 | SMN |  |

Note: The corresponding full names of the brain regions are in the supplementary material. The number of analyzed in each group is: n(MCI vs. NC)=78, n(AD vs. NC)=135, n(AD vs.MCI)=76, n(sMCI vs. pMCI)=26.

Abbreviations: AN, attention network; DMN, default mode network; SMN, sensorimotor network; SUBN, subcortical network; VN, visual network.

### Imaging marker identification and validation

**Table S7:** Overview of previous state-of-art GCN studies distinguishing between individuals with AD and cognitively normal subjects.

| **Study** | **group** | **Sample size** | **Accuracy** | **AUC** |
| --- | --- | --- | --- | --- |
| J. Lee et al., 2021 | CN vs. MCI | 101 | 0.74 | 0.74 |
| Z. Qin et al., 2020 | CN vs. AD | 91 | 0.83 | NA |
| P. Gu et al., 2021 | CN vs. AD | 216 | 0.95 | 0.95 |
| L. Mei et al., 2022 | CN vs. MCI | 483 | 0.73 | NA |
| A. Kumar et al., 2022 | CN vs. AD | 189 | ≈0.78 | NA |
| H. Tang et al., 2022 | CN vs. AD | 1326 | 0.78 | NA |

### The features of the important brain regions predict the clinical progression MCI to AD


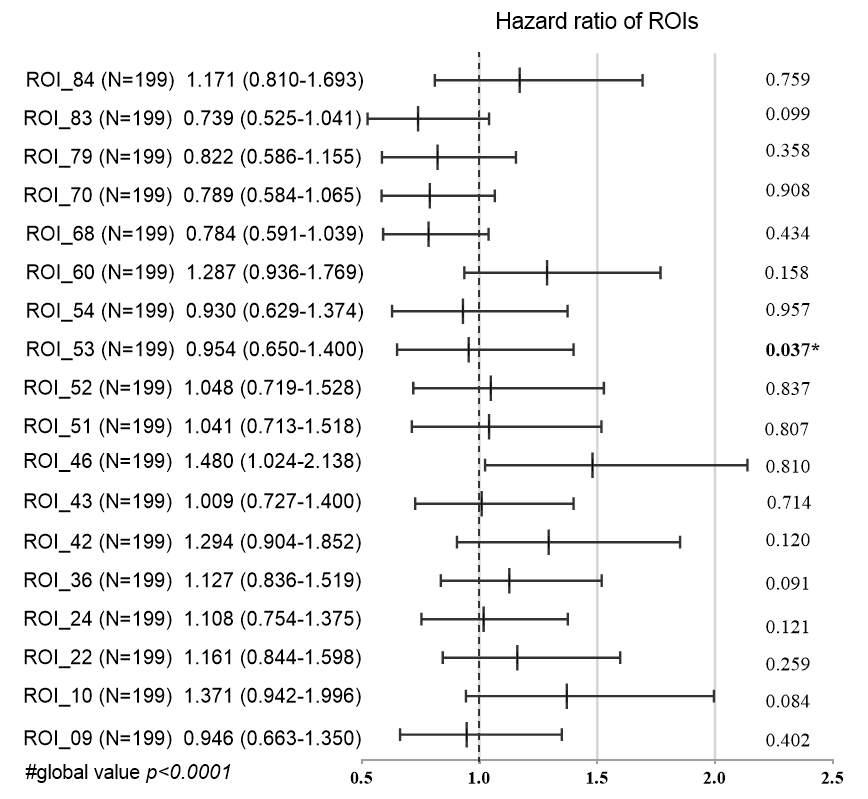


**Figure S6:** Hazard ratios for different predictors. These predictors are the pathological brain regions found in this study based on the STGC-GCAM model. The leftmost part of the figure is the corresponding brain area number and its risk value (95% confidence interval), and the right side is the p value of the predictor, and *p*<0.05 is considered significant.

### Causal mediation analyses


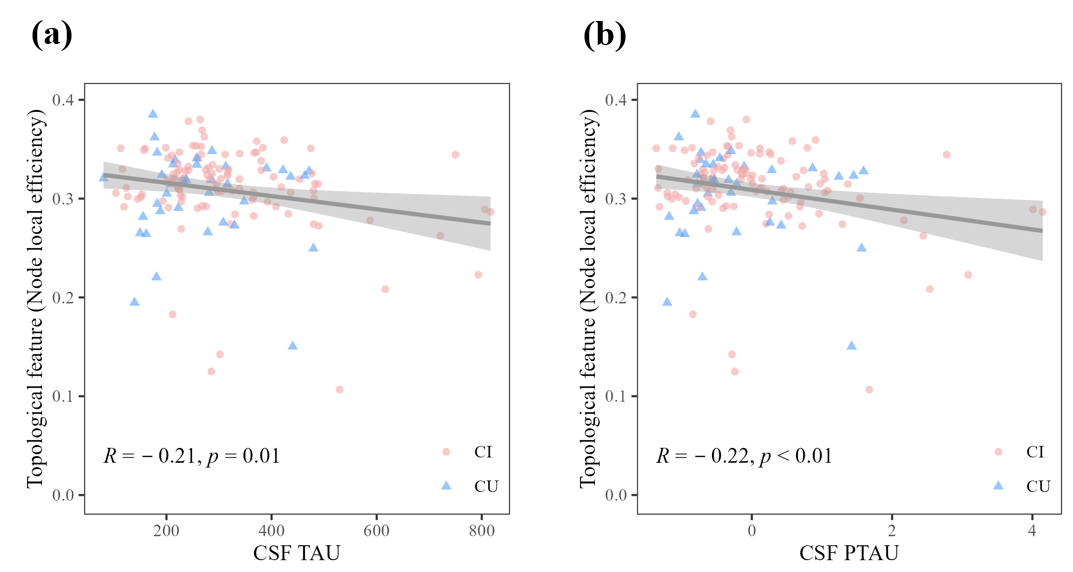


**Figure S7:** Topological features of important brain regions were correlated with biomarkers. (a) the node local efficiency of the important brain region and CSF TAU are significantly correlated (R = -0.21, p = 0.01). (b) the node local efficiency of the important brain region and CSF PTAU are significantly correlated (R = -0.22, p < 0.01).

## References

[1] C. Sheng *et al.*, "Combining Visual Rating Scales for Medial Temporal Lobe Atrophy and Posterior Atrophy to Identify Amnestic Mild Cognitive Impairment from Cognitively Normal Older Adults: Evidence Based on Two Cohorts," *J Alzheimers Dis,* vol. 77, no. 1, pp. 323-337, 2020.

[2] G. M. McKhann *et al.*, "The diagnosis of dementia due to Alzheimer's disease: recommendations from the National Institute on Aging-Alzheimer's Association workgroups on diagnostic guidelines for Alzheimer's disease," *Alzheimers Dement,* vol. 7, no. 3, pp. 263-9, May 2011.
